# Supplementary material for: Modeling serological testing to inform relaxation of social distancing for COVID-19 control
Source: Nat Commun. 2021 Dec 3;12:7063. doi: 10.1038/s41467-021-26774-y (PMC8642547; doi:10.1038/s41467-021-26774-y)
Supplement: Supplementary file 1 — Supplementary Information [file 41467_2021_26774_MOESM1_ESM.pdf]

Supplementary Material for : Modeling serological testing to inform relaxation of social distancing for COVID-19 control

Alicia N. M. Kraay[1,a], Kristin N. Nelson[1,a], Conan Zhao[2,3], David Demory[2], Joshua S. Weitz[2,4,b], Benjamin A. Lopman[1,b]

[1] Rollins School of Public Health, Emory University, Atlanta, GA, USA
[2] School of Biological Sciences, Georgia Institute of Technology, Atlanta, GA, USA
[3] Interdisciplinary Graduate Program in Quantitative Biosciences, Georgia Institute of Technology, Atlanta, GA, USA
[4] School of Physics, Georgia Institute of Technology, Atlanta, GA, USA
October 18, 2021

S1. Model Equations

The ordinary differential equations describing the model are shown below for group i. The positive test group is denoted with '+'.

[a]These authors contributed equally to this work
[b]These authors contributed equally to this work

$$
\begin{aligned}
\frac{dS_i}{dt} &= -\lambda_i(t)S_i - (1-\mathrm{f}) \times \mathrm{t}_i(t)S_i \\
\frac{dE_i}{dt} &= \lambda_i(t)S_i - \gamma_e E_i - (1-\mathrm{f}) \times \mathrm{t}_i(t)E_i \\
\frac{dI_{s,i}}{dt} &= \gamma_e E_i p - \gamma_s I_{s,i} \\
\frac{dI_{a,i}}{dt} &= \gamma_e E_i(1-p) - \gamma_a I_{a,i} - (1-\mathrm{f}) \times \mathrm{t}_i(t)I_{a,i} \\
\frac{dH_{s,i}}{dt} &= \gamma_s I_{s,i}(\mathrm{H}_i - \mathrm{r}_i) + \gamma_s I^+_{s,i}(\mathrm{H}_i - \mathrm{r}_i) - \gamma_{hs} H_{s,i} \\
\frac{dH_{c,i}}{dt} &= \gamma_s I_{s,i}\mathrm{r}_i + \gamma_s I^+_{s,i}\mathrm{r}_i - \gamma_{\mathrm{hc}} H_{\mathrm{c,i}} \\
\frac{dD_i}{dt} &= \gamma_{\mathrm{hc}} H_{\mathrm{c,i}}\mathrm{d}_i \\
\frac{dR_i}{dt} &= (1-\mathrm{H}_i)\gamma_s I_{s,i} + \gamma_a I_{a,i} + (1-\mathrm{s}) \times m_2(t)\gamma_{\mathrm{hs}} H_{s,i} + (1-\mathrm{s}) \times m_2(t)\gamma_{hc} H_{c,i}(1-\mathrm{d}_i) \\
&\quad - \mathrm{s} \times \mathrm{t}_i(t)R_i + m_1(t)\gamma_{\mathrm{hs}} H_{s,i} + m_1(t)\gamma_{hc} H_{c,i}(1-\mathrm{d}_i) \\
\frac{dS^+_i}{dt} &= (1-\mathrm{f})\mathrm{t}_i(t)S_i - \lambda^+_i(t)S^+_i \\
\frac{dE^+_i}{dt} &= (1-\mathrm{f})\mathrm{t}_i(t)E_i + \lambda^+_i(t)S^+_i - \gamma_e E^+_i \\
\frac{dI^+_{s,i}}{dt} &= \gamma_e E^+_i p - \gamma_s I^+_{s,i} \\
\frac{dI^+_{a,i}}{dt} &= (1-\mathrm{f}) \times \mathrm{t}_i(t)I_{a,i} + \gamma_e E^+_i(1-p) - \gamma_a I^+_{a,i} \\
\frac{dR^+_i}{dt} &= \mathrm{s} \times \mathrm{t}_i(t)R_i + s\gamma_{hc} m_2(t)H^+_{c,i}(1-\mathrm{d}_i) + \\
&\mathrm{s} \times m_2(t)\gamma_{hs} H_{s,i} + (1-\mathrm{H}_i)\gamma_s I^+_{s,i} + \gamma_a I^+_{a,i}
\end{aligned}
$$

$m_1(t)$ and $m_2(t)$ are defined as follows (t_{start} is when testing starts, on November 1, 2020):

$$
m_1(t) = \begin{cases} 1 & t < t_{\text{start}} \\ 0 & t \geq t_{\text{start}} \end{cases}
$$

$$
m_2(t) = \begin{cases} 0 & t < t_{\text{start}} \\ 1 & t \geq t_{\text{start}} \end{cases}
$$

The force of infection $\lambda(t)$ for the i-th group is a function of the number of social contacts for age group i with each subgroup j at time t ($x_{i,j}(t)$), the probability of infection given contact (q), the number of infections in each group at time t ($\text{infec}_j(t)$), and the population size of each group at time t ($n_j(t)$). The overall equation for $\lambda_i(t)$ is shown below:

$$\lambda_i(t) = q\Bigg[\frac{x_{i,ch}(t)\mathrm{i}_{ch}(t)}{n_{ch}(t)} + \frac{x_{i,ch^+}(t)\mathrm{i}_{ch^+}(t)}{n_{ch^+}(t)} + \frac{x_{i,ad}(t)\mathrm{i}_{ad}(t)}{n_{ad}(t)} + \frac{x_{i,ad^+}(t)\mathrm{i}_{ad^+}(t)}{n_{ad^+}(t)} + \frac{x_{i,rc}(t)\mathrm{i}_{rc}(t)}{n_{rc}(t)} + \frac{x_{i,rc^+}(t)\mathrm{i}_{rc^+}(t)}{n_{rc^+}(t)} + \frac{x_{i,fc}(t)\mathrm{i}_{fc}(t)}{n_{fc}(t)} + \frac{x_{i,fc^+}(t)\mathrm{i}_{fc^+}(t)}{n_{fc^+}(t)} + \frac{x_{i,el}(t)\mathrm{i}_{el}(t)}{n_{el}(t)} + \frac{x_{i,el^+}(t)\mathrm{i}_{el^+}(t)}{n_{el^+}(t)}\Bigg]$$

i and j take values of ch (children age 0-19 years), ad (adults age 20-65 years who are not working or working from home), el (older adults 65+ years of age), rc (adults at work in 'reduced-contact' occupations, where they have fewer contacts than pre-pandemic), fc (adults as work in full contact occupations, where they have the same number of contacts as pre-pandemic).

The number of infectious individuals by age group and test status is equal to the sum of documented (symptomatic) cases and a fraction of the undocumented (asymptomatic) cases, where this fraction η corresponds to the relative infectiousness of undocumented cases. This is shown below for children:

$$\mathrm{i}_{ch}(t) = \eta I_{\text{a,ch}} + I_{\text{s,ch}}$$

S2. Contact matrices

Baseline contacts

Baseline contact matrices for 'work' contacts, 'school' contacts, 'home' contacts, and 'other' contacts were taken from [1]. To expand these baseline matrices to the 5 population groups in our model (separating the adult population into fc, rc, and h classes), we multiplied all contacts with adults $x_{i,ad}$ by the proportion of the adult population falling into each class. We define the fraction of the population falling in each working group as follows:

$$f_{home} = \frac{n_{\text{home}}}{n_{\text{home}} + n_{\text{reduced}} + n_{\text{full}}}$$
$$f_{reduced} = \frac{n_{\text{reduced}}}{n_{\text{home}} + n_{\text{reduced}} + n_{\text{full}}}$$
$$f_{full} = \frac{n_{\text{full}}}{n_{\text{home}} + n_{\text{reduced}} + n_{\text{full}}}$$

For baseline contact matrix values based on [1], we have:

$$x_{i,j} = \begin{bmatrix} x_{ch,ch} & x_{ch,ad} & x_{ch,el} \\ x_{ad,ch} & x_{ad,ad} & x_{ad,el} \\ x_{el,ch} & x_{el,ad} & x_{el,el} \end{bmatrix}$$

For simplicity, we assume that baseline interactions between worker subgroups are only assortative with respect to age (and not with respect to occupation type). To expand this matrix to a 5x5 matrix we use the following notation, where rows 2, 3, and 4 correspond to the work from home, reduced contact, and full contact occupation groups, respectively:

$$x_{i,j} = \begin{bmatrix} x_{ch,ch} & x_{ch,ad}f_{home} & x_{ch,ad}f_{reduced} & x_{ch,ad}f_{full} & x_{ch,el} \\ x_{ad,ch} & x_{ad,ad}f_{home} & x_{ad,ad}f_{reduced} & x_{ad,ad}f_{full} & x_{ch,el} \\ x_{ad,ch} & x_{ad,ad}f_{home} & x_{ad,ad}f_{reduced} & x_{ad,ad}f_{full} & x_{ch,el} \\ x_{ad,ch} & x_{ad,ad}f_{home} & x_{ad,ad}f_{reduced} & x_{ad,ad}f_{full} & x_{ch,el} \\ x_{el,ch} & x_{el,ad}f_{home} & x_{el,ad}f_{reduced} & x_{el,ad}f_{full} & x_{ch,el} \end{bmatrix}$$

Based on these proportions, we define $x_{i,h}$, $x_{i,rc}$, and $x_{i,fc}$ as follows:

$$\begin{aligned} x_{i,h} &= x_{i,ad}f_{home} \\ x_{i,rc} &= x_{i,ad}f_{reduced} \\ x_{i,fc} &= x_{i,ad}f_{full} \end{aligned}$$

Contacts under social distancing

After social distancing has begun, we assume that:

- Home contacts remain the same.
- Schools close.
- Only working age adults continue to work. Baseline workplace contacts for children and young adults under 20 years of age are nearly zero (average 0.84 contacts/day) and the average workplace contacts for the elderly is 0, so this does not appreciably impact our results.
- All working adults who are able work from home.
- Adults continuing to work outside the home reduce their workplace contacts by constant $p_{reduced}$.
- Other contacts are reduced by scalar constant θ_o.

The revised contact matrix for work contacts then becomes:

$$M_{work} = \begin{bmatrix} 0 & 0 & 0 & 0 & 0 \\ 0 & 0 & 0 & 0 & 0 \\ x_{ad,ch}\theta_w & x_{h,h}\theta_w & x_{h,rc}\theta_w & x_{h,fc}\theta_w & x_{ch,el}\theta_w \\ x_{fc,ch} & x_{fc,h} & x_{fc,rc} & x_{fc,fc} & x_{ch,el} \\ 0 & 0 & 0 & 0 & 0 \end{bmatrix}$$

The revised contact matrix for other contacts becomes:

$$M_{other} = \theta_o \times \begin{bmatrix} x_{ch,ch} & x_{ch,h} & x_{ch,rc} & x_{ch,fc} & x_{ch,el} \\ x_{h,ch} & x_{h,h} & x_{h,rc} & x_{h,fc} & x_{ch,el} \\ x_{rc,ch} & x_{rc,h} & x_{rc,rc} & x_{rc,f} & x_{ch,el} \\ x_{fc,ch} & x_{fc,h} & x_{fc,rc} & x_{fc,fc} & x_{ch,el} \\ x_{el,ch} & x_{el,h} & x_{el,rc} & x_{el,fc} & x_{ch,el} \end{bmatrix}$$

Contacts during initial relaxing of social distancing

When stay at home orders are initially lifted, we assume that:

- Home contacts remain the same.
- Adults who were working from home continue to work from home.
- Workers in reduced contact occupations increase their workplace contacts based on the intensity of social distancing maintained.
- Schools remain closed until September 1, 2020 in South Florida and until October 1, 2020 in New York City and Washington, after which time they reopen at 50% capacity.
- Other contacts continue to be reduced based on the intensity of social distancing maintained.

Contacts after testing begins

When testing begins, we assume that:

- Test-positive individuals move to the test positive group.
- Home contacts remain the same, but their distribution by test status is driven by the proportion of test-positives in the general population.
- Adults who were working from home may return to work if they test positive. Upon returning to work, their workplace contacts are assortative with respect to test status (but not with respect to occupation type).
- Workers in reduced contact occupations increase their workplace contacts based on the intensity of social distancing maintained. Work contacts are preferentially with test-positive individuals, as determined by α, or shielding strength.
- Other contacts are increased for test positive individuals to their pre-pandemic levels. Other contacts continue to be reduced for test negative/untested individuals based on the intensity of social distancing maintained. Other contacts are preferentially with test-positive individuals, as determined by α, or shielding strength.

After testing has begun, all contact matrices are dependent on the proportion of the population that has tested positive and been released from social distancing at time t. We define this proportion as $r_i(t)$, where $(1 - r_i(t))$ is the fraction of the population who has not yet tested positive.

We assume that social distancing parameters are relaxed from their initial values as follows:

$$\theta_{o2} = 1 - ((1 - \theta_o) \times c)$$
$$\theta_{w2} = 1 - ((1 - \theta_w) \times c)$$

For contact matrices of work and 'other' contacts, we implement shielding factor α, which increases the probability of contacting a test-positive individual according to their prevalence in the population (achieved by multiplying expected contact rates due to prevalence by scaling factor $\alpha + 1$). To account for the fact that, when prevalence is high, $(\alpha + 1)r_i(t)$ may exceed 1, we introduce a variable $s_i(t)$:

$$s_i(t) = \begin{cases} (\alpha + 1)r_i(t) & (\alpha + 1)r_i(t) \leq 1 \\ 1 & (\alpha + 1)r_i(t) \geq 1 \end{cases}$$

This shielding structure is similar to 'fixed shielding' [2] in that it preserves the baseline number of contacts and increases contacts for test positive individuals by $1 + \alpha$, as shown below:

$$x_0 = x_0 r_i(t) + x_0(1 - r_i(t))$$
$$x_0 = r_i(t)(\alpha + 1)x_0 + (x_0 - (\alpha + 1)r_i(t)x_0)$$

The structure of all three matrices (home, work, and other) is given by M:

$$M_{home} = \begin{bmatrix} r_{ch}(t)x_{ch,ch} & (1-r_{ch}(t))x_{ch,ch} & r_h(t)x_{ch,h} & (1-r_h(t))x_{ch,h} & r_{rc}(t)x_{ch,rc} & (1-r_{rc}(t))x_{ch,rc} & r_{fc}(t)x_{ch,fc} & (1-r_{fc}(t))x_{ch,fc} & r_{el}(t)x_{ch,el} & (1-r_{el}(t))x_{ch,el} \\ r_{ch}(t)x_{ch,ch} & (1-r_{ch}(t))x_{ch,ch} & r_h(t)x_{ch,h} & (1-r_h(t))x_{ch,h} & r_{rc}(t)x_{ch,rc} & (1-r_{rc}(t))x_{ch,rc} & r_{fc}(t)x_{ch,fc} & (1-r_{fc}(t))x_{ch,fc} & r_{el}(t)x_{ch,el} & (1-r_{el}(t))x_{ch,el} \\ r_{ch}(t)x_{h,ch} & (1-r_{ch}(t))x_{h,ch} & r_h(t)x_{h,h} & (1-r_h(t))x_{h,h} & r_{rc}(t)x_{h,rc} & (1-r_{rc}(t))x_{h,rc} & r_{fc}(t)x_{h,fc} & (1-r_{fc}(t))x_{h,fc} & r_{el}(t)x_{h,el} & (1-r_{el}(t))x_{h,el} \\ r_{ch}(t)x_{h,ch} & (1-r_{ch}(t))x_{h,ch} & r_h(t)x_{h,h} & (1-r_h(t))x_{h,h} & r_{rc}(t)x_{h,rc} & (1-r_{rc}(t))x_{h,rc} & r_{fc}(t)x_{h,fc} & (1-r_{fc}(t))x_{h,fc} & r_{el}(t)x_{h,el} & (1-r_{el}(t))x_{h,el} \\ r_{ch}(t)x_{rc,ch} & (1-r_{ch}(t))x_{rc,ch} & r_h(t)x_{rc,h} & (1-r_h(t))x_{rc,h} & r_{rc}(t)x_{rc,rc} & (1-r_{rc}(t))x_{rc,rc} & r_{fc}(t)x_{rc,fc} & (1-r_{fc}(t))x_{rc,fc} & r_{el}(t)x_{rc,el} & (1-r_{el}(t))x_{rc,el} \\ r_{ch}(t)x_{rc,ch} & (1-r_{ch}(t))x_{rc,ch} & r_h(t)x_{rc,h} & (1-r_h(t))x_{rc,h} & r_{rc}(t)x_{rc,rc} & (1-r_{rc}(t))x_{rc,rc} & r_{fc}(t)x_{rc,fc} & (1-r_{fc}(t))x_{rc,fc} & r_{el}(t)x_{rc,el} & (1-r_{el}(t))x_{rc,el} \end{bmatrix}$$

$$M_{other} = \begin{bmatrix} 1 \\ \theta_{o2} \\ 1 \\ \theta_{o2} \\ 1 \\ \theta_{o2} \\ 1 \\ \theta_{o2} \\ 1 \\ \theta_{o2} \end{bmatrix} \times \begin{bmatrix} s_{ch}(t)x_{ch,ch} & (1-s_{ch}(t))x_{ch,ch} & s_h(t)x_{ch,h} & (1-s_h(t))x_{ch,h} & s_{rc}(t)x_{ch,rc} & (1-s_{rc}(t))x_{ch,rc} & s_{fc}(t)x_{ch,fc} & (1-s_{fc}(t))x_{ch,fc} & s_{el}(t)x_{ch,el} & (1-s_{el}(t))x_{ch,el} \\ s_{ch}(t)x_{ch,ch} & (1-s_{ch}(t))x_{ch,ch} & s_h(t)x_{ch,h} & (1-s_h(t))x_{ch,h} & s_{rc}(t)x_{ch,rc} & (1-s_{rc}(t))x_{ch,rc} & s_{fc}(t)x_{ch,fc} & (1-s_{fc}(t))x_{ch,fc} & s_{el}(t)x_{ch,el} & (1-s_{el}(t))x_{ch,el} \\ s_{ch}(t)x_{h,ch} & (1-s_{ch}(t))x_{h,ch} & s_h(t)x_{h,h} & (1-s_h(t))x_{h,h} & s_{rc}(t)x_{h,rc} & (1-s_{rc}(t))x_{h,rc} & s_{fc}(t)x_{h,fc} & (1-s_{fc}(t))x_{h,fc} & s_{el}(t)x_{h,el} & (1-s_{el}(t))x_{h,el} \\ s_{ch}(t)x_{h,ch} & (1-s_{ch}(t))x_{h,ch} & s_h(t)x_{h,h} & (1-s_h(t))x_{h,h} & s_{rc}(t)x_{h,rc} & (1-s_{rc}(t))x_{h,rc} & s_{fc}(t)x_{h,fc} & (1-s_{fc}(t))x_{h,fc} & s_{el}(t)x_{h,el} & (1-s_{el}(t))x_{h,el} \\ s_{ch}(t)x_{rc,ch} & (1-s_{ch}(t))x_{rc,ch} & s_h(t)x_{rc,h} & (1-s_h(t))x_{rc,h} & s_{rc}(t)x_{rc,rc} & (1-s_{rc}(t))x_{rc,rc} & s_{fc}(t)x_{rc,fc} & (1-s_{fc}(t))x_{rc,fc} & s_{el}(t)x_{rc,el} & (1-s_{el}(t))x_{rc,el} \\ s_{ch}(t)x_{rc,ch} & (1-s_{ch}(t))x_{rc,ch} & s_h(t)x_{rc,h} & (1-s_h(t))x_{rc,h} & s_{rc}(t)x_{rc,rc} & (1-s_{rc}(t))x_{rc,rc} & s_{fc}(t)x_{rc,fc} & (1-s_{fc}(t))x_{rc,fc} & s_{el}(t)x_{rc,el} & (1-s_{el}(t))x_{rc,el} \\ s_{ch}(t)x_{fc,ch} & (1-s_{ch}(t))x_{fc,ch} & s_h(t)x_{fc,h} & (1-s_h(t))x_{fc,h} & s_{rc}(t)x_{fc,rc} & (1-s_{rc}(t))x_{fc,rc} & s_{fc}(t)x_{fc,fc} & (1-s_{fc}(t))x_{fc,fc} & s_{el}(t)x_{fc,el} & (1-s_{el}(t))x_{fc,el} \\ s_{ch}(t)x_{fc,ch} & (1-s_{ch}(t))x_{fc,ch} & s_h(t)x_{fc,h} & (1-s_h(t))x_{fc,h} & s_{rc}(t)x_{fc,rc} & (1-s_{rc}(t))x_{fc,rc} & s_{fc}(t)x_{fc,fc} & (1-s_{fc}(t))x_{fc,fc} & s_{el}(t)x_{fc,el} & (1-s_{el}(t))x_{fc,el} \\ s_{ch}(t)x_{el,ch} & (1-s_{ch}(t))x_{el,ch} & s_h(t)x_{el,h} & (1-s_h(t))x_{el,h} & s_{rc}(t)x_{el,rc} & (1-s_{rc}(t))x_{el,rc} & s_{fc}(t)x_{el,fc} & (1-s_{fc}(t))x_{el,fc} & s_{el}(t)x_{el,el} & (1-s_{el}(t))x_{el,el} \\ s_{ch}(t)x_{el,ch} & (1-s_{ch}(t))x_{el,ch} & s_h(t)x_{el,h} & (1-s_h(t))x_{el,h} & s_{rc}(t)x_{el,rc} & (1-s_{rc}(t))x_{el,rc} & s_{fc}(t)x_{el,fc} & (1-s_{fc}(t))x_{el,fc} & s_{el}(t)x_{el,el} & (1-s_{el}(t))x_{el,el} \end{bmatrix}$$

$$M_{work} = \begin{bmatrix} 0 \\ 0 \\ 1 \\ 0 \\ \theta_{w2} \\ \theta_{w2} \\ 1 \\ 1 \\ 0 \\ 0 \end{bmatrix} \times \begin{bmatrix} 0 & 0 & 0 & 0 & 0 & 0 & 0 & 0 & 0 & 0 \\ 0 & 0 & 0 & 0 & 0 & 0 & 0 & 0 & 0 & 0 \\ x_{h,ch} & 0 & x_{h,h} & 0 & x_{h,rc} & 0 & x_{h,fc} & 0 & x_{h,el} & 0 \\ 0 & 0 & 0 & 0 & 0 & 0 & 0 & 0 & 0 & 0 \\ s_{ch}(t)x_{rc,ch} & (1-s_{ch}(t))x_{rc,ch} & s_h(t)x_{rc,h} & (1-s_h(t))x_{rc,h} & s_{rc}(t)x_{rc,rc} & (1-s_{rc}(t))x_{rc,rc} & s_{fc}(t)x_{rc,fc} & (1-s_{fc}(t))x_{rc,fc} & s_{el}(t)x_{rc,el} & (1-s_{el}(t))x_{rc,el} \\ s_{ch}(t)x_{rc,ch} & (1-s_{ch}(t))x_{rc,ch} & s_h(t)x_{rc,h} & (1-s_h(t))x_{rc,h} & s_{rc}(t)x_{rc,rc} & (1-s_{rc}(t))x_{rc,rc} & s_{fc}(t)x_{rc,fc} & (1-s_{fc}(t))x_{rc,fc} & s_{el}(t)x_{rc,el} & (1-s_{el}(t))x_{rc,el} \\ 0 & 0 & 0 & 0 & 0 & 0 & 0 & 0 & 0 & 0 \\ 0 & 0 & 0 & 0 & 0 & 0 & 0 & 0 & 0 & 0 \end{bmatrix}$$

S3. Model parameters

We defined the three metropolitan areas in the same way as Havers et al [3]. For Washington Puget Sound metropolitan region, we included death data from King, Snohomish, Pierce, Kitsap and Grays Harbor counties. For the New York City metropolitan region, we included data from Manhattan, Bronx, Queens, Kings, and Nassau Counties. For the South Florida metropolitan region, we included data from Miami-Dade, Broward, Palm Beach, and Martin counties. These same counties were also used to derive age-specific population sizes for each region.

Parameters used in the model simulations are shown in Table 1 and Table 2. We assume that the size of the working population is stable over the duration of the simulation. Although this may not be the case as unemployment increases throughout the pandemic, the rate at which unemployment has changed so far has been time-varying and its future trajectory is unknown.

Where possible, parameters were taken from prior literature. We fixed all parameters in Table 1 as well as the site-specific population age structure and the timing of public health interventions. We did not vary these parameters as part of our fitting procedure–only fitted parameters were considered to be uncertain as part of our fitting procedure and it is the uncertainty in these parameters that is captured by the credible intervals. We fitted the following parameters: the probability of infection per contact (q), the strength of social distancing (c), the fraction of infections symptomatic (p), the initial intensity of social distancing for workplace contacts (θ_w) and for other contacts (θ_o). These parameters were fitted to initial outbreak dynamics from each of the three metropolitan locations. While q does not vary with time, we acknowledge that some control measures such as masking may result in changes in the probability of infection given contact over the course of the pandemic. In our model, changes in the contact matrix based on both the initial strength of social distancing (θ_w and θ_o) and its strength after reopening captures the contribution of both reducing the number of social contacts and changes in the likelihood of transmission from those contacts based on mask use and physical distancing.

We used the dates that stay-at-home orders were enacted, and later lifted, in each location and the dates corresponding to when local schools opened for at least partial in-person instruction in each location to inform dates of reopening in the model.

S4. Model fitting

Initial Conditions

We first calculate the number of weeks between the first death and the first week where the cumulative death toll exceeds 10. For example, in the South Florida region, the first death was reported the week of March 18, 2020. The two subsequent weeks saw the death toll rise to 6 and 42. Using region-specific conditions (population demographics, stay-at-home order and lift dates, and deaths data), we initialize an epidemic consisting of a single exposed adult ($a0$). We use baseline estimates for the parameters we aim to fit later (q = 0.0451, c = 1, $p = 0.14$, $\theta_o = 0.25$, $\theta_w = 0.1$). We forward simulate a single-origin epidemic until the modeled number of deaths exceeds the number of deaths reported in the second week after the first death (in South Florida, 42 deaths), then use the distribution two weeks prior as our initial conditions.

Parameter	Code	Value	Units	Source(s)
**Natural history**				
Latent period	$1/\gamma_e$	1/3	1/days	[4]
Recovery rate, asymptomatic infections	γ_a	1/7	1/days	[5]
Recovery rate, symptomatic infections	γ_s	1/7	1/days	[5]
Recovery rate, hospitalized cases	γ_{hs}	1/5	1/days	[6]
Recovery rate, critical care cases	γ_{hc}	1/7	1/days	[6]
Relative infectiousness of asymptomatic infections	η	0.55	–	[7, 8]
**Hospitalization**				
Probability of hospitalization				[9]
Children	H_{ch}	0.061	–	
Adults	H_{ad}	0.182	–	
Elderly	H_{el}	0.417	–	
Probability of requiring critical care				[9]
Children	r_{ch}	0	–	
Adults	r_{ad}	0.063	–	
Elderly	r_{el}	0.173	–	
Probability of death among critical care patients				
Children	d_{ch}	0	–	[10]
Adults	d_{ad}	0.5	–	[7]
Elderly	d_{el}	0.5	–	[7]
**Test features**				
Sensitivity	s	1.00	–	[11]
Specificity	f	0.998 (0.5, 0.998)	–	[11]
**Population features**				
Population size				[12]
New York City		9.43e6	people	
South Florida		6.17e6	people	
Washington Puget Sound		4.06e6	people	
Baseline contact rates by age	$x_{i,j}$	see matrices		[1]
Adult working population segments, size				[13]
Exclusive work from home occupations	n_{home}	$0.316 \times n_{adult}$	people	
Reduced contact occupations	$n_{reduced}$	$0.628 \times n_{adult}$	people	
Full contact occupations	n_{full}	$0.057 \times n_{adult}$	people	
Fraction of adult population (20-65 years) in workforce		1.0		assumption
**Intervention parameters**				
Shielding	α	(0, 9)	per contact	varied
Fraction tested	$\mathrm{t}_i(t)$	(0, 0.03)	proportion per day	varied

Supplementary Table 1: Fixed parameters across locations used in model simulations. Values in parentheses show where values were varied across simulations to reflect different testing and shielding scenarios.

Parameter	Code	New York	South Florida	Washington	Units	Source(s)
**Fitted parameters**						
Fraction symptomatic [1],[2]	p	0.310	0.140	0.410	–	fitted
		(0.26,0.35)	(0.08,0.19)	(0.29,0.54)		
Probability of infection per contact [3]	q	0.042	0.050	0.026	1/contact	fitted
		(0.041, 0.043)	(0.049,0.052)	(0.024,0.028)		
Strength of social distancing maintained[1]	c	0.366	0.800	0.156	–	fitted
		(0.002,0.96)	(0.54,0.98)	(0.001,0.66)		
Fraction of work contacts maintained (rc workers)[1],[4]	θ_w	0.641	0.241	0.202	–	fitted
		(0.37, 0.88)	(0.001, 0.74)	(0.002, 0.77)		
Fraction of 'other' contacts maintained [1],[5]	θ_o	0.028	0.120	0.130	–	fitted
		(0.001, 0.14)	(0.002, 0.38)	(0.001, 0.43)		
Epidemic start date	t_0	Feb 20	Feb 27	Feb 13	date	fitted
Basic reproduction number	R_0	2.99	3.24	1.99	–	calculated from fitted values
**Population features (fixed)**						
Baseline contact rates by age	$x_{i,j}$		see matrices			[1]
US population age groups, size						[14, 12]
Children	n_{child}	2208909	1398572	974924	people	
Adults	n_{adult}	5944919	3679331	2563244	people	
Elderly	$n_{elderly}$	1283369	1090711	526665	people	
**Intervention timing (fixed)**						
Stay at home order start	–	Mar 16	Mar 17	Mar 16	date	[15, 16, 17]
Reopening	–	June 8	May 20	May 31	date	[15, 16, 17]
School reopening	–	Oct 1	Sept 1	Oct 1	date	assumption

[1] Constrained so fitted value would fall between 0 and 1

[2] Note that p is equivalent to $symptomatic\_fraction$ in Supplementary Figures 2-9

[3] Constrained so fitted value would fall between 0 and 0.07

[4] Note that θ_w is equivalent to $p\_reduced$ in Supplementary Figures 2-9

[5] Note that θ_o is equivalent to $socialDistancing\_other$ in Supplementary Figures 2-9

Supplementary Table 2: Location-specific parameters used in model simulations. Values shown for fitted parameters are the maximum likelihood estimate, used for main text simulations in Figures 2 and 3, and values in parentheses represent the 95% CI used to calculate simulation intervals

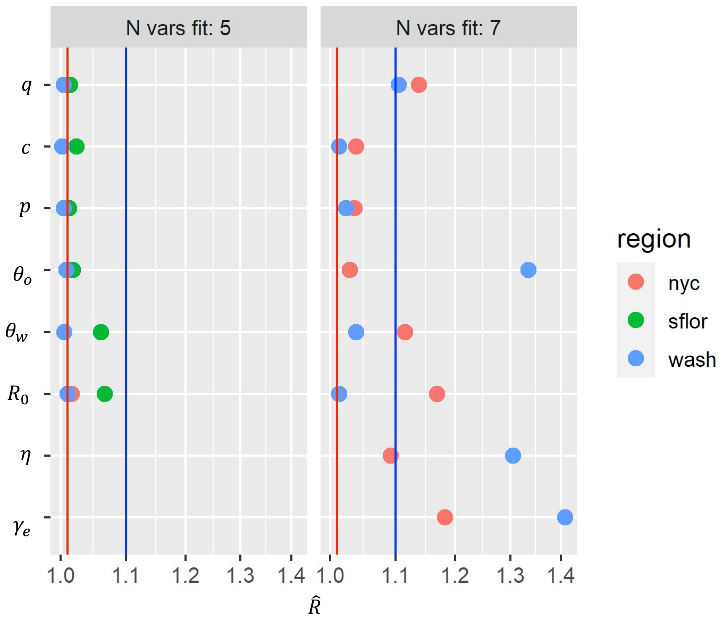

Supplementary Figure 1: Gelman-Rubin diagnostics plot. We calculate Gelman-Rubin $\widehat{R}$ values for chain convergence among all 10 chains for all three sites. $\widehat{R}$ values for all parameters were below 1.1 (blue vertical line), with the majority under the 1.01 convergence threshold (red vertical line). [18]

MCMC

Using Markov Chain Monte Carlo (MCMC) we estimate the five model parameters listed in Table 2 with uniform priors on a fixed range (q bounded from 0 to 0.07; c, p, θ_o, and θ_w bounded from 0 to 1).

The penalized log-likelihood function is defined as follows: let $d_o(t)$ be the observed cumulative number of deaths at time t and $r_o(t)$ be the observed weekly death rate. Let $d_s(t)$ be the model simulated cumulative number of deaths and $r_s(t)$ be the weekly death rates at time t. Let t_m and t_f denote a midpoint (11 weeks) and final time point of our simulation window. Let σ_s and σ_o denote the simulated and observed population-level seroprevalence estimates for each location by *Havers et al*[3]. Let $a = 100000$ be a scaling factor and F_{lp} denote the Poisson log-likelihood function. The log-likelihood function (LL)is defined as:

$$LL = \sum_t \left(F_{lp}\left(\frac{r_s(t)}{a}, \frac{r_o(t)}{a} \right) \right) + F_{lp}\left(\frac{d_s(t_m)}{a}, \frac{d_o(t_m)}{a} \right) + F_{lp}\left(\frac{d_s(t_f)}{a}, \frac{d_o(t_f)}{a} \right) + F_{lp}\left(\frac{\sigma_s}{a}, \frac{\sigma_o}{a} \right)$$

We checked for chain convergence using the Gelman-Rubin diagnostic (Supplementary Figure 1). We also considered fitting the relative transmissibility of asymptomatic cases (η) and the latent period ($1/\gamma_e$) (7 parameter fits in Supplementary Figure 1) but these fits did not converge and were therefore not considered further. Supplementary Figures 2, 5, and 8 show the resulting trace plots, and Supplementary Figures 3, 6, and 9 show the resulting joint distributions.

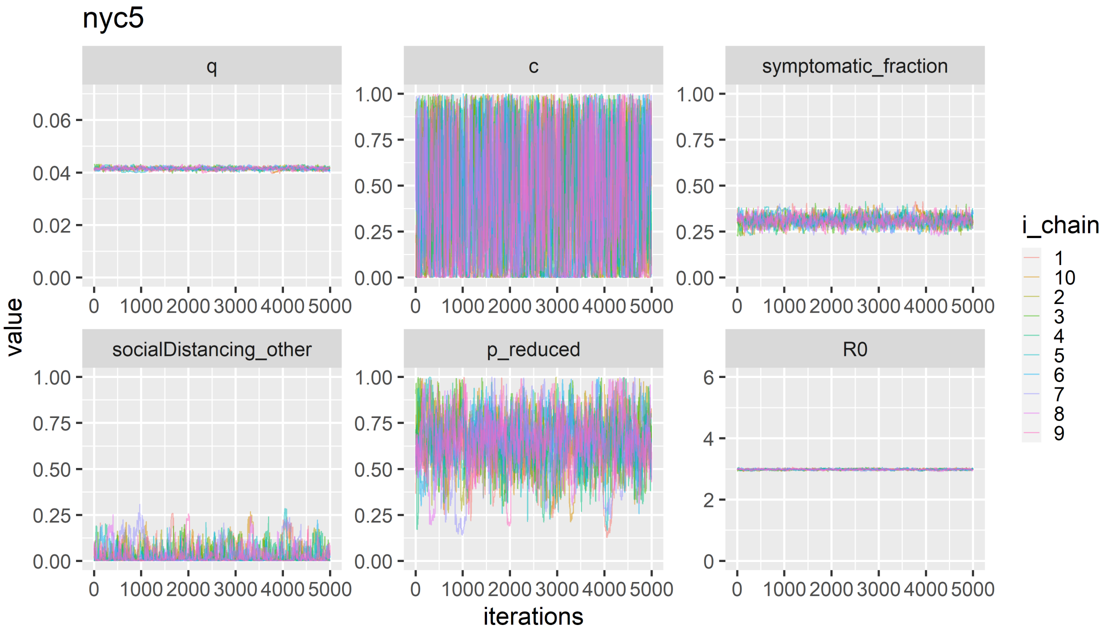

Supplementary Figure 2: **New York City MCMC Traceplots.** The last 5,000 iterations from each MCMC chain is plotted. Y-axis ranges are limited to show parameter search space constraints. R_0 traces are calculated from q and $symptomatic\_fraction$ traces.

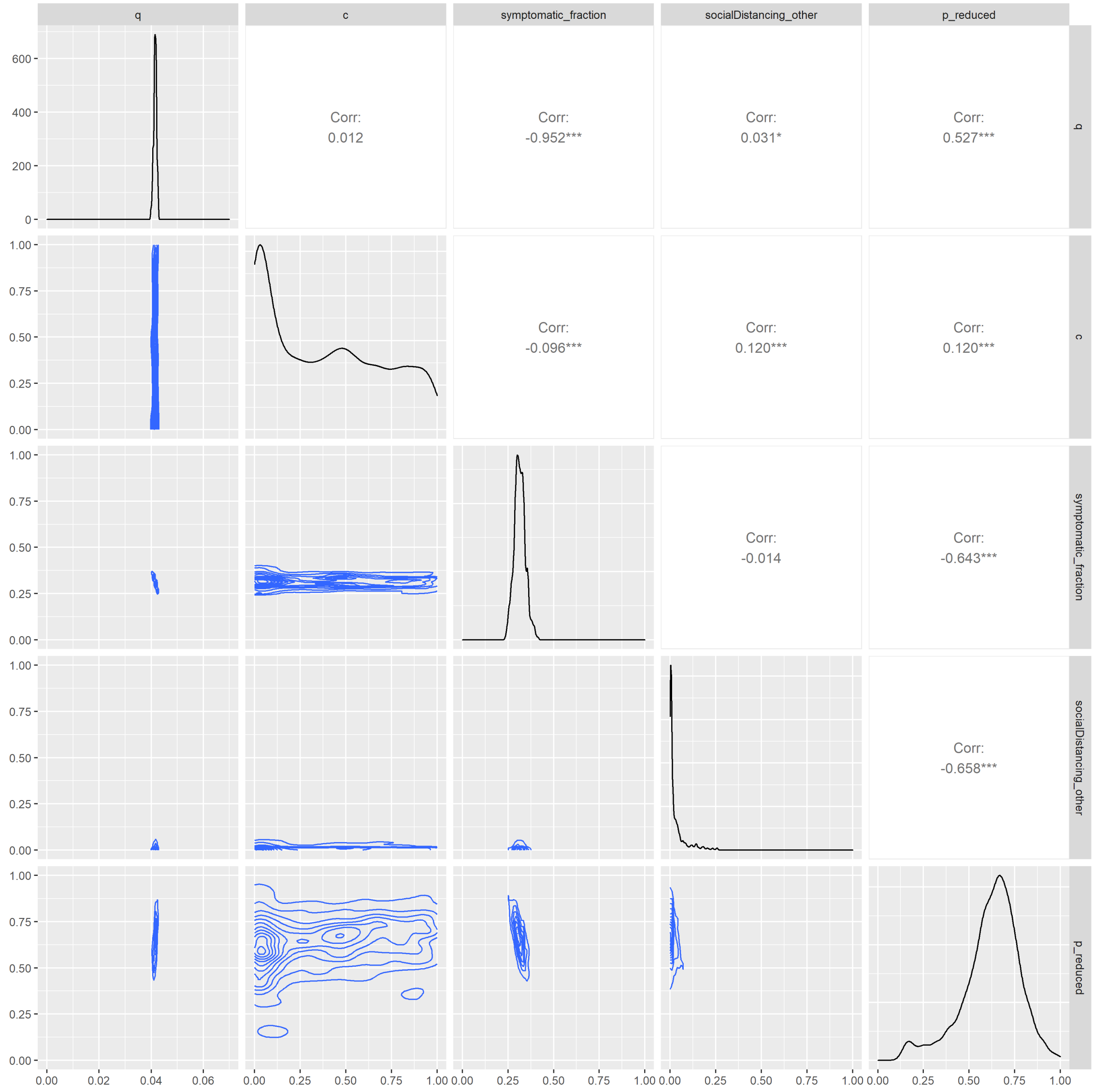

Supplementary Figure 3: **New York City Pairwise Parameter Correlations.** Joint posterior distributions for pairs of parameters fitted to data from the New York City region for weekly reported deaths and seroprevalence estimates. Represented is chain 1. Two-sided p-values are shown for the pairwise Pearson correlation coefficients. The asterisks indicate the significance levels of the correlations. Each significance level is associated to a symbol : p-values(0, 0.001, 0.01, 0.05, 0.1, 1) ¡=¿ symbols("***", "**", "*", ".", " ") For example, '**' indicates the correlation is significant at the 0.001 level. (two-tailed)

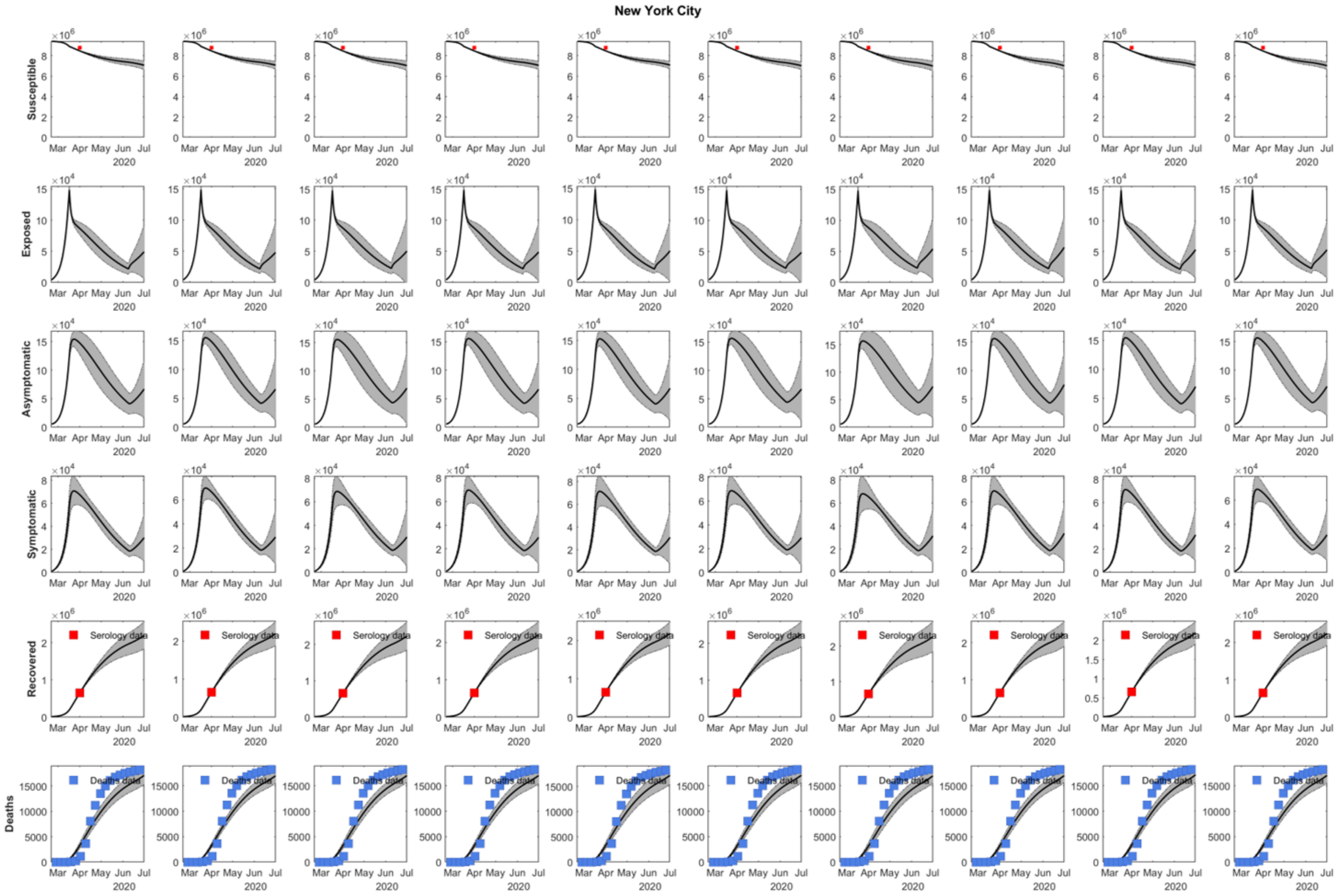

Supplementary Figure 4: **New York City Model Fits**. We forward simulate a model using the maximum likelihood estimates of each parameter (black) and 100 random parameter set draws from the posterior distribution (gray). Simulated data are presented as mean (black line) +/- 1.96 sd (grey bands). Cumulative deaths are plotted (blue squares), in addition to seroprevalence estimates (red squares) in each location. Each column corresponds to an MCMC chain. Each chain is seeded randomly.

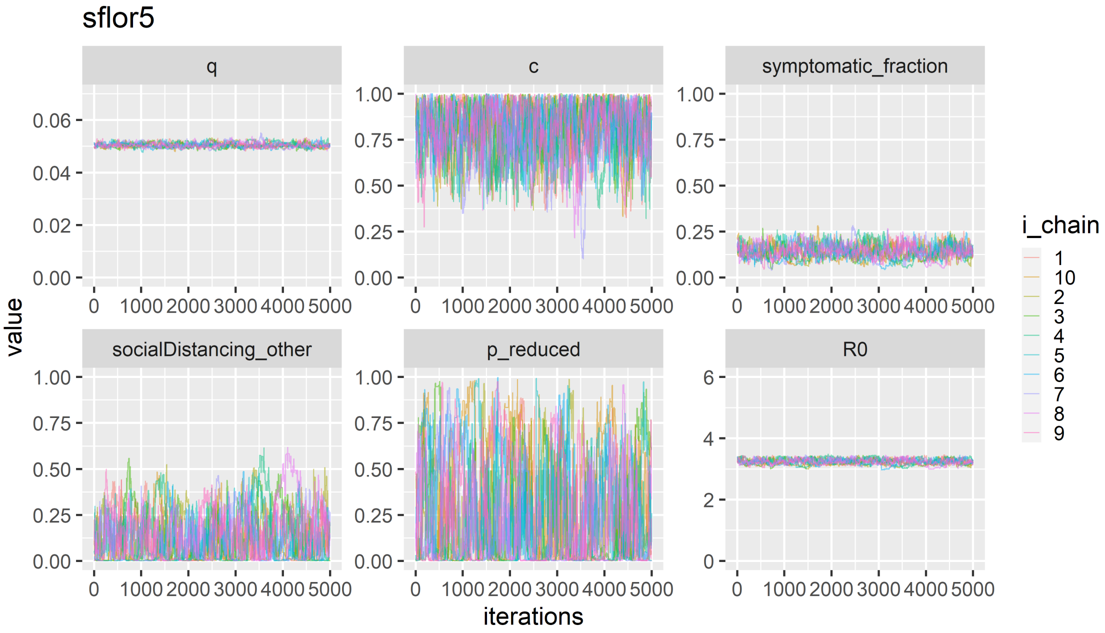

Supplementary Figure 5: **South Florida Post-burn-in MCMC Traceplots.** The last 5,000 iterations from each MCMC chain is plotted. Y-axis ranges are limited to show parameter search space constraints. R_0 traces are calculated from q and $symptomatic\_fraction$ traces.

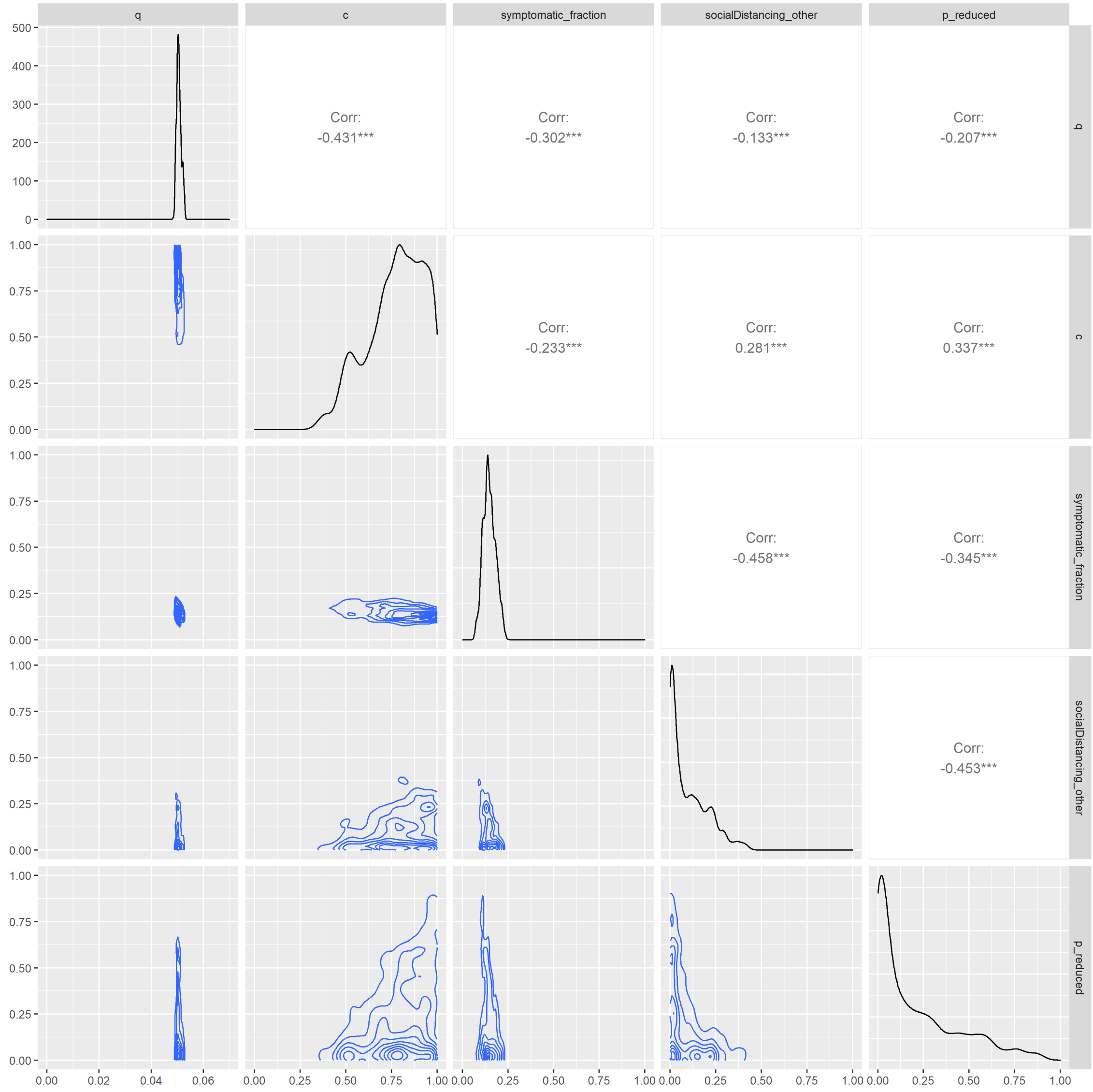

Supplementary Figure 6: **South Florida Pairwise Parameter Correlations.** Joint posterior distributions for pairs of parameters fitted to data from the South Florida region for weekly reported deaths and seroprevalence estimates. Represented is chain 1. Two-sided p-values are shown for the pairwise Pearson correlation coefficients. The asterisks indicate the significance levels of the correlations. Each significance level is associated to a symbol : p-values(0, 0.001, 0.01, 0.05, 0.1, 1) ¡=¿ symbols(“***”, “**”, “*”, “.”, ” ”) For example, ’**’ indicates the correlation is significant at the 0.001 level. (two-tailed)

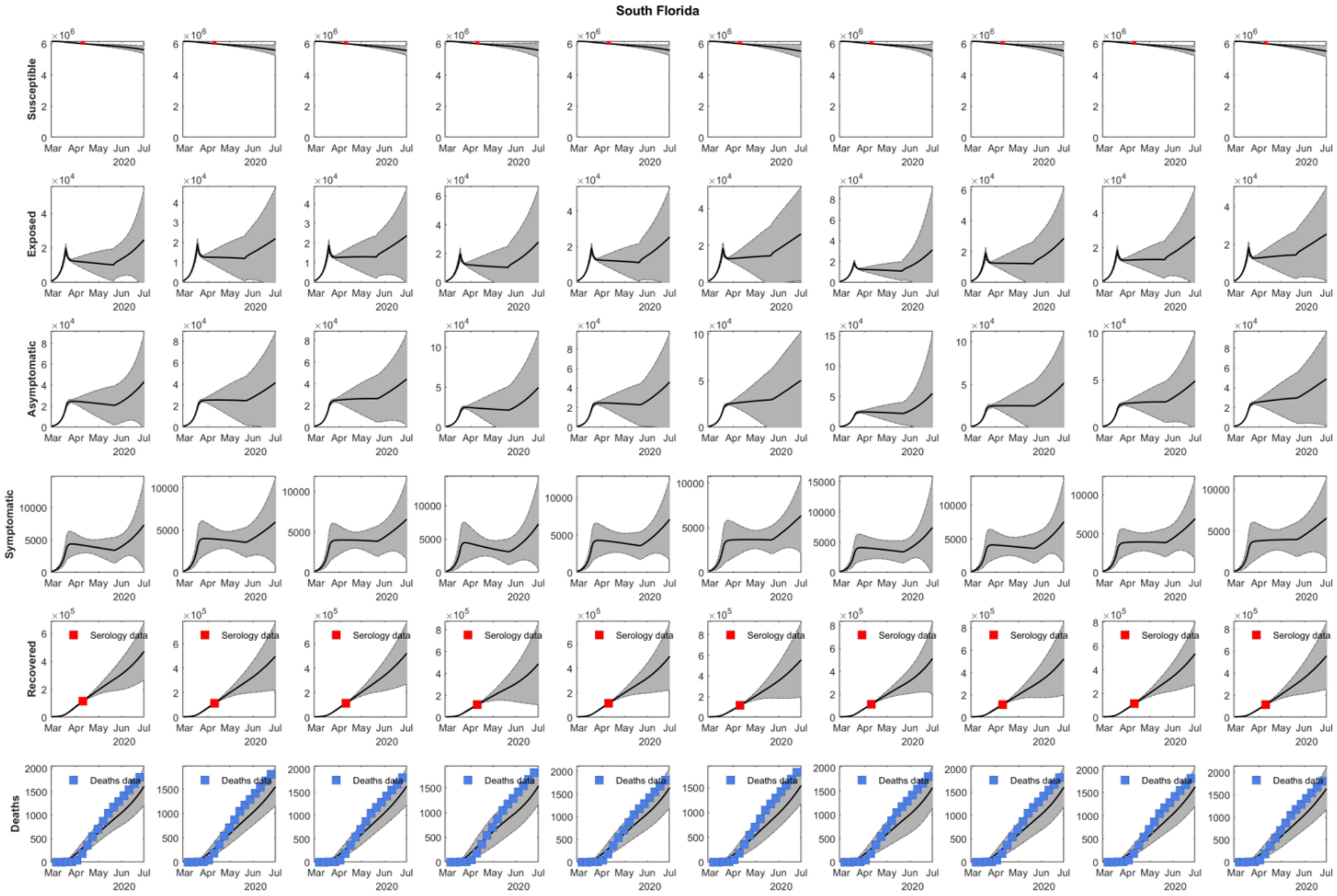

Supplementary Figure 7: **South Florida Model Fits.** We forward simulate a model using the maximum likelihood estimates of each parameter (black) and 100 random parameter set draws from the posterior distribution (gray). Simulated data are presented as mean (black line) +/- 1.96 sd (grey bands). Cumulative deaths are plotted (blue squares), in addition to seroprevalence estimates (red squares) in each location. Each column corresponds to an MCMC chain. Each chain is seeded randomly.

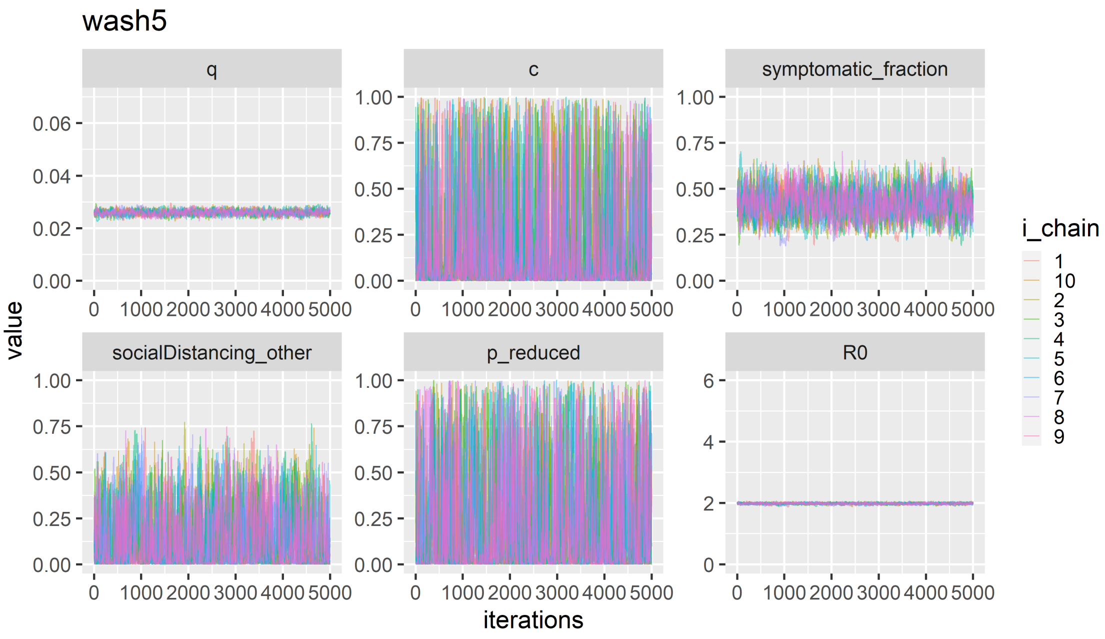

Supplementary Figure 8: **Washington Puget Sound Post-burn-in MCMC Traceplots.** The last 5,000 iterations from each MCMC chain is plotted. Y-axis ranges are limited to show parameter search space constraints. R_0 traces are calculated from q and $symptomatic\_fraction$ traces.

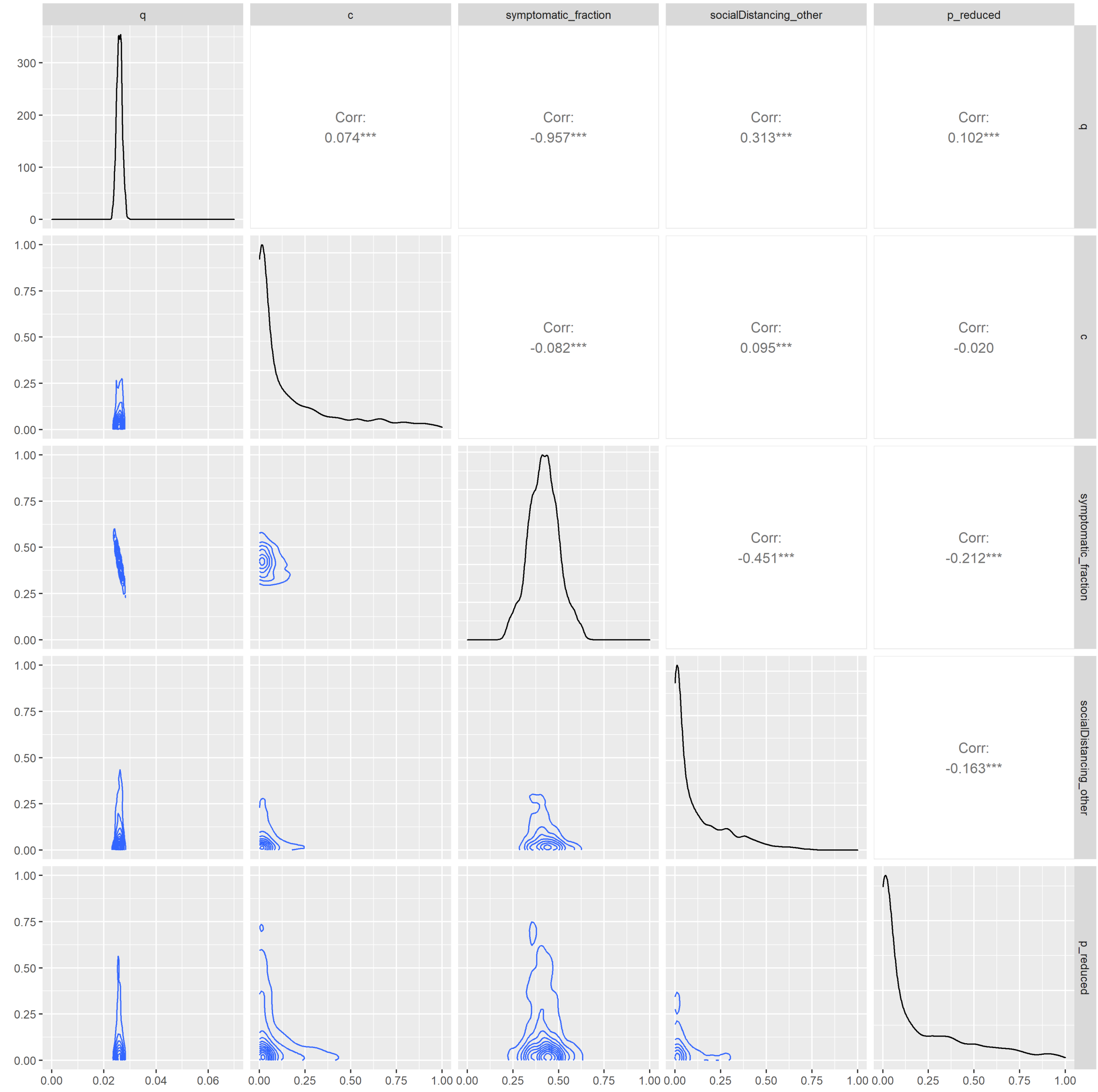

Supplementary Figure 9: **Washington Pairwise Parameter Correlations.** Joint posterior distributions for pairs of parameters fitted to data from the Washington Puget Sound region for weekly reported deaths and seroprevalence estimates. Represented is chain 1. Two-sided p-values are shown for the pairwise Pearson correlation coefficients. The asterisks indicate the significance levels of the correlations. Each significance level is associated to a symbol : p-values(0, 0.001, 0.01, 0.05, 0.1, 1) ¡=¿ symbols(“***”, “**”, “*”, “.”, ” ”) For example, ’**’ indicates the correlation is significant at the 0.001 level. (two-tailed)

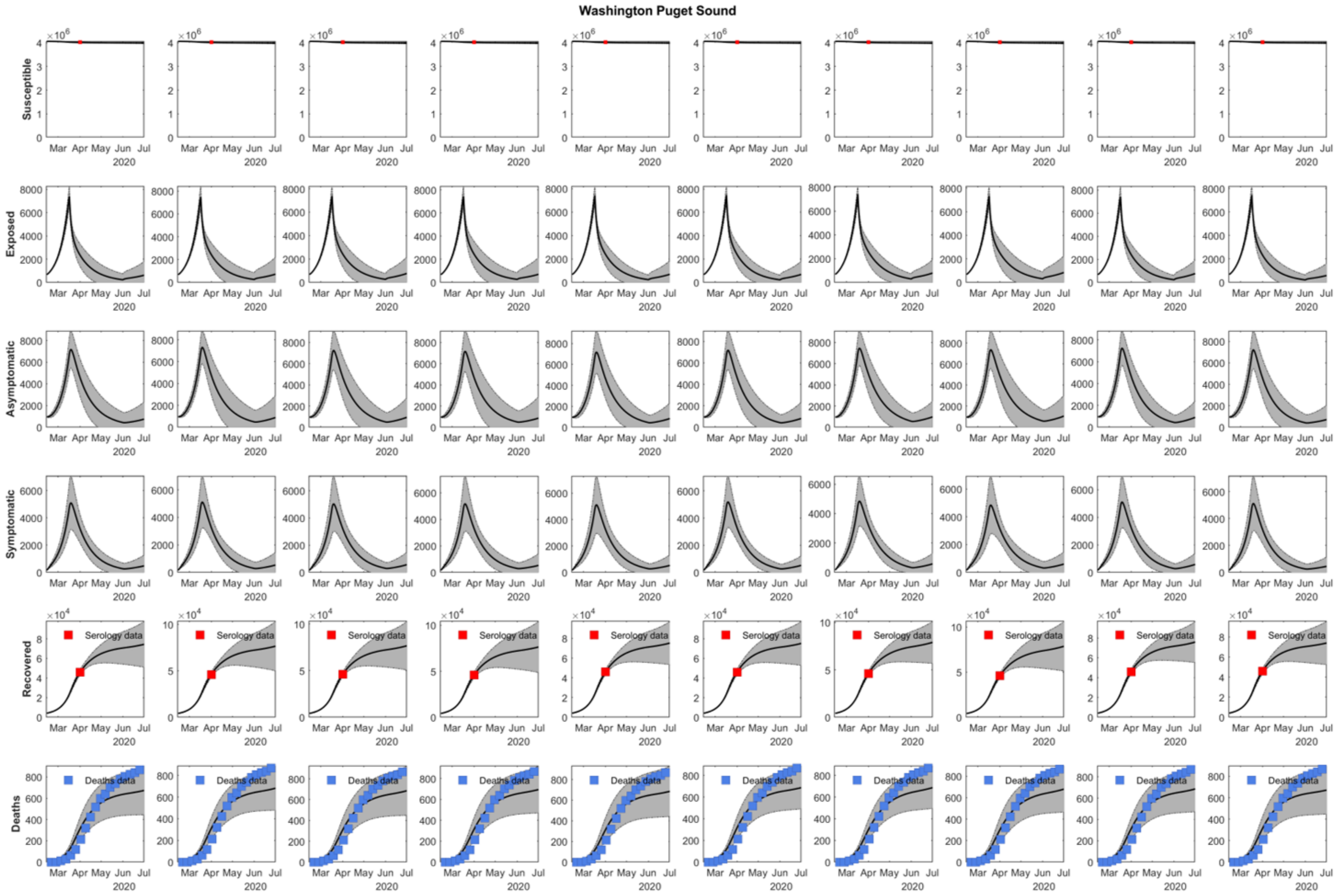

Supplementary Figure 10: **Washington Model Fits.** We forward simulate a model using the maximum likelihood estimates of each parameter (black) and 100 random parameter set draws from the posterior distribution (gray). Simulated data are presented as mean (black line) +/- 1.96 sd (grey bands). Cumulative deaths are plotted (blue squares), in addition to seroprevalence estimates (red squares) in each location. Each column corresponds to an MCMC chain. Each chain is seeded randomly.

Credible intervals width

The overall width of credible intervals (as shown in the main text) was determined by the value of c (Supplementary Figure 14). As shown in the trace plots, the value of c was not identifiable from our model simulations, particularly for Washington and New York City, with a wide variety of initial values matching the initial dynamics. In South Florida, the fitted value of c was more constrained, yet model predictions still in large part depended on the ongoing level of social distancing. In part, this issue arose because the relaxation of social distancing in these two locations began after the first wave of the epidemic was largely complete (main text Figure 3), and thus there were few cases shortly after reopening with which to calibrate the dynamics. However, as the outbreak continued, this parameter was crucial for determining the number of deaths by the time shielding was implemented, and hence its ultimate impact. While adding to the length of the time series of deaths used to fit the model might have improved identifiability, ultimately the level of ongoing social distancing is likely to be highly time-varying. The width of the credible interval thus reflects the importance of ongoing social distancing to determine both the trajectory of the United States epidemic and the potential impact of any other control interventions.

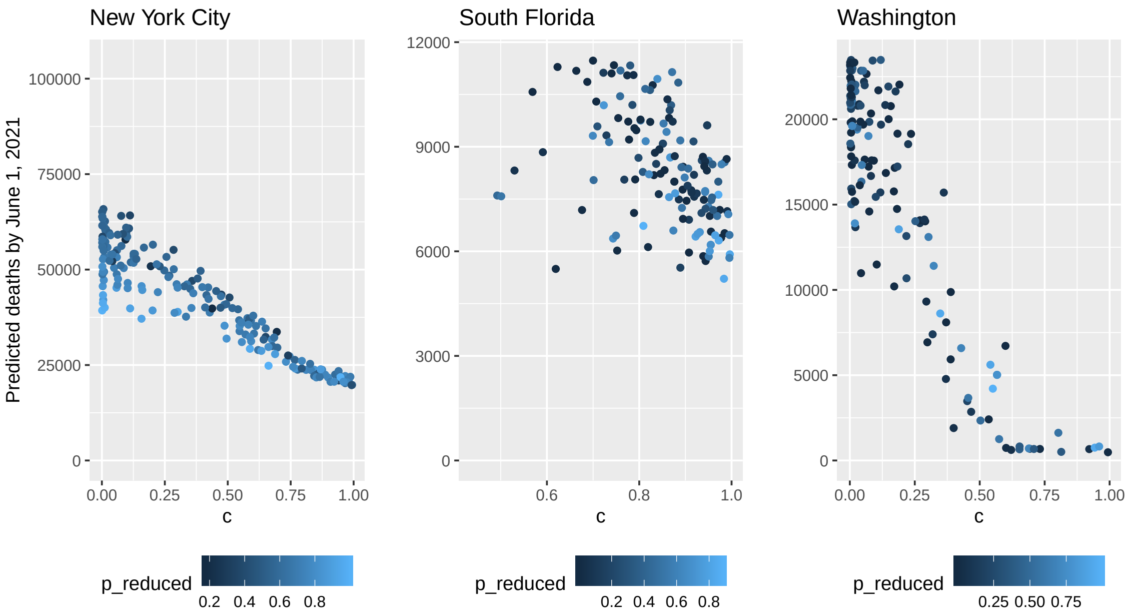

Supplementary Figure 11: Number of deaths expected after 1 year (y-axis) for each setting (panels) without testing as a function of the degree of relaxation of social distancing (given by c, shown on the x-axis) and initial social distancing strength (colors indicate the value of θ_w) ($p_{reduced}$).

S5. R_0 Estimation

The dynamics of the system and R_0 are determined by how the outbreak would proceed at time zero in the absence of any interventions, and therefore depends on the fitted values of p and q, but not on any of the other fitted parameters. Therefore, we assume no testing at time 0, no social distancing, and no differences in worker contact levels (i.e., all groups mix at the population-average level prior to the outbreak). In this situation, there are only 3 population subgroups at time zero:

$$
\begin{aligned}
\frac{dE_{ch}}{dt} &= \lambda_{ch} S_{ch} - \gamma_e E_{ch} \\
\frac{dI_{s,ch}}{dt} &= \gamma_e E_{ch} p - \gamma_s I_{s,ch} \\
\frac{dI_{a,ch}}{dt} &= \gamma_e E_{ch}(1-p) - \gamma_a I_{a,ch} \\
\frac{dH_{s,ch}}{dt} &= \gamma_s I_{s,ch}(\mathrm{H}_{ch} - \mathrm{r}_{ch}) - \gamma_{hs} \mathrm{H}_{s,ch} \\
\frac{dH_{c,ch}}{dt} &= \gamma_s I_{s,ch} \mathrm{r}_{ch} - \gamma_{hc} \mathrm{H}_{c,ch} \\
\frac{dE_{ad}}{dt} &= \lambda_{ad} S_{ad} - \gamma_e E_{ad} \\
\frac{dI_{s,ad}}{dt} &= \gamma_e E_{ad} p - \gamma_s I_{s,ad} \\
\frac{dI_{a,ad}}{dt} &= \gamma_e E_{ad}(1-p) - \gamma_a I_{a,ad} \\
\frac{dH_{s,ad}}{dt} &= \gamma_s I_{s,ad}(\mathrm{H}_{ad} - \mathrm{r}_{ad}) - \gamma_{hs} H_{s,ad} \\
\frac{dH_{c,ad}}{dt} &= \gamma_s I_{s,ad} \mathrm{r}_{ad} - \gamma_{hc} \mathrm{H}_{c,ad} \\
\frac{dE_{el}}{dt} &= \lambda_{el} S_{el} - \gamma_e E_{el} \\
\frac{dI_{s,el}}{dt} &= \gamma_e E_{el} p - \gamma_s I_{s,el} \\
\frac{dI_{a,el}}{dt} &= \gamma_e E_{el}(1-p) - \gamma_a I_{a,el} \\
\frac{dH_{s,el}}{dt} &= \gamma_s I_{s,el}(\mathrm{H}_{el} - \mathrm{r}_{el}) - \gamma_{hs} \mathrm{H}_{s,el} \\
\frac{dH_{c,el}}{dt} &= \gamma_s I_{s,el} \mathrm{r}_{el} - \gamma_{hc} \mathrm{H}_{c,el}
\end{aligned}
$$

The λ_i for each group is defined as follows.

$$
\lambda_{ch} = \frac{q x_{ch,ch}(\eta I_{a,ch} + I_{s,ch})}{n_{ch}} + \frac{q x_{ch,ad}(\eta I_{a,ad} + I_{s,ad})}{n_{ad}} + \frac{q x_{ch,el}(\eta I_{a,el} + I_{s,el})}{n_{el}}
$$

$$
\lambda_{ad} = \frac{q x_{ad,ch}(\eta I_{a,ch} + I_{s,ch})}{n_{ch}} + \frac{q x_{ad,ad}(\eta I_{a,ad} + I_{s,ad})}{n_{ad}} + \frac{q x_{ad,el}(\eta I_{a,el} + I_{s,el})}{n_{el}}
$$

$$\lambda_{el} = \frac{qx_{el,ch}(\eta I_{a,ch} + I_{s,ch})}{n_{ch}} + \frac{qx_{el,ad}(\eta I_{a,ad} + I_{s,ad})}{n_{ad}} + \frac{qx_{el,el}(\eta I_{a,el} + I_{s,el})}{n_{el}}$$

The matrices $\mathcal{F}$ and $\mathcal{V}$ corresponding to these equations are:

$$
\mathcal{F} = \begin{bmatrix}
0 & qx_{ch,ch} & qx_{ch,ch}\eta & 0 & 0 & 0 & qc_{ch,ad} & qx_{ch,ad}\eta & 0 & 0 & 0 & qx_{ch,el} & qx_{ch,el}\eta & 0 & 0 \\
0 & 0 & 0 & 0 & 0 & 0 & 0 & 0 & 0 & 0 & 0 & 0 & 0 & 0 & 0 \\
0 & 0 & 0 & 0 & 0 & 0 & 0 & 0 & 0 & 0 & 0 & 0 & 0 & 0 & 0 \\
0 & 0 & 0 & 0 & 0 & 0 & 0 & 0 & 0 & 0 & 0 & 0 & 0 & 0 & 0 \\
0 & 0 & 0 & 0 & 0 & 0 & 0 & 0 & 0 & 0 & 0 & 0 & 0 & 0 & 0 \\
0 & qx_{ad,ch} & qx_{ad,ch}\eta & 0 & 0 & 0 & qx_{ad,ad} & qx_{ad,ad}\eta & 0 & 0 & 0 & qx_{ad,el} & qx_{ad,el}\eta & 0 & 0 \\
0 & 0 & 0 & 0 & 0 & 0 & 0 & 0 & 0 & 0 & 0 & 0 & 0 & 0 & 0 \\
0 & 0 & 0 & 0 & 0 & 0 & 0 & 0 & 0 & 0 & 0 & 0 & 0 & 0 & 0 \\
0 & 0 & 0 & 0 & 0 & 0 & 0 & 0 & 0 & 0 & 0 & 0 & 0 & 0 & 0 \\
0 & 0 & 0 & 0 & 0 & 0 & 0 & 0 & 0 & 0 & 0 & 0 & 0 & 0 & 0 \\
0 & qx_{el,ch} & qx_{el,ch}\eta & 0 & 0 & 0 & qx_{el,ad} & qx_{el,ad}\eta & 0 & 0 & 0 & qx_{el,el} & qx_{el,el}\eta & 0 & 0 \\
0 & 0 & 0 & 0 & 0 & 0 & 0 & 0 & 0 & 0 & 0 & 0 & 0 & 0 & 0 \\
0 & 0 & 0 & 0 & 0 & 0 & 0 & 0 & 0 & 0 & 0 & 0 & 0 & 0 & 0 \\
0 & 0 & 0 & 0 & 0 & 0 & 0 & 0 & 0 & 0 & 0 & 0 & 0 & 0 & 0 \\
0 & 0 & 0 & 0 & 0 & 0 & 0 & 0 & 0 & 0 & 0 & 0 & 0 & 0 & 0
\end{bmatrix}
$$

$$
\mathcal{V} = \begin{bmatrix}
\gamma_e & 0 & 0 & 0 & 0 & 0 & 0 & 0 & 0 & 0 & 0 & 0 & 0 & 0 & 0 \\
-\gamma_e p & \gamma_s & 0 & 0 & 0 & 0 & 0 & 0 & 0 & 0 & 0 & 0 & 0 & 0 & 0 \\
-\gamma_e(1-p) & 0 & \gamma_a & 0 & 0 & 0 & 0 & 0 & 0 & 0 & 0 & 0 & 0 & 0 & 0 \\
0 & -\gamma_s(\mathrm{H}_{ch} - \mathrm{r}_{ch}) & 0 & \gamma_{hs} & 0 & 0 & 0 & 0 & 0 & 0 & 0 & 0 & 0 & 0 & 0 \\
0 & -\gamma_s \mathrm{r}_{ch} & 0 & 0 & \gamma_{hc} & 0 & 0 & 0 & 0 & 0 & 0 & 0 & 0 & 0 & 0 \\
0 & 0 & 0 & 0 & 0 & \gamma_e & 0 & 0 & 0 & 0 & 0 & 0 & 0 & 0 & 0 \\
0 & 0 & 0 & 0 & 0 & -\gamma_e p & \gamma_s & 0 & 0 & 0 & 0 & 0 & 0 & 0 & 0 \\
0 & 0 & 0 & 0 & 0 & -\gamma_e(1-p) & 0 & \gamma_a & 0 & 0 & 0 & 0 & 0 & 0 & 0 \\
0 & 0 & 0 & 0 & 0 & 0 & -\gamma_s(\mathrm{H}_{ad} - \mathrm{r}_{ad}) & 0 & \gamma_{hs} & 0 & 0 & 0 & 0 & 0 & 0 \\
0 & 0 & 0 & 0 & 0 & 0 & -\gamma_s \mathrm{r}_{ad} & 0 & 0 & \gamma_{hc} & 0 & 0 & 0 & 0 & 0 \\
0 & 0 & 0 & 0 & 0 & 0 & 0 & 0 & 0 & 0 & \gamma_e & 0 & 0 & 0 & 0 \\
0 & 0 & 0 & 0 & 0 & 0 & 0 & 0 & 0 & 0 & -\gamma_e p & \gamma_s & 0 & 0 & 0 \\
0 & 0 & 0 & 0 & 0 & 0 & 0 & 0 & 0 & 0 & -\gamma_e(1-p) & 0 & \gamma_a & 0 & 0 \\
0 & 0 & 0 & 0 & 0 & 0 & 0 & 0 & 0 & 0 & 0 & 0 & -\gamma_s(\mathrm{H}_{el} - \mathrm{r}_{el} & \gamma_{hs} & 0 \\
0 & 0 & 0 & 0 & 0 & 0 & 0 & 0 & 0 & 0 & 0 & 0 & -\gamma_s \mathrm{r}_{el} & 0 & \gamma_{hc}
\end{bmatrix}
$$

To derive an expression for R_0 we inverted the $\mathcal{V}$ matrix and multiplied by $\mathcal{F}$. The dominant eigenvalues of this matrix can be computed, but are very complex and are therefore not shown here. It is notable that because hospitalized cases do not contribute to the force of infection, the value of R_0 does not depend on γ_{hs} or γ_{hc}. We calculated the value of R_0 for each of the three locations based on the fitted values of p and q and the other relevant parameters.

S6. Overall contact reductions by location

Location	Reduction in work contacts for adults	Reduction in other contacts for all groups	Reduction in total contacts		
			Children	Adults	Elderly
New York City	39.8%	35.6%	37.5%	30.0%	20.9%
South Florida	69.7%	70.4%	44.8%	53.2%	40.3%
Washington	39.4%	13.6%	26.5%	18.6%	6.9%

Supplementary Table 3: Steady state reduction in contacts by location after stay-at-home orders are lifted and schools reopen

S7. Sensitivity of model results to fixed parameters

Overall PRCC results are shown in Supplementary Figures 12-14. In general, increased relative transmissibility for asymptomatic cases tended to increase the potential for deaths averted through testing/shielding, whereas faster recovery rates for both symptomatic and asymptomatic cases tended to decrease the potential impact of shielding. These patterns were most pronounced for South Florida and Washington. In New York City, a shorter latent period was associated with decreased potential impact of shielding when monthly testing with a highly specific test was used, but not in any of the other intervention scenarios considered. These patterns were reversed when testing was employed without shielding. The duration of hospitalization did not alter the ultimate impact of a shielding strategy in any of the sites.

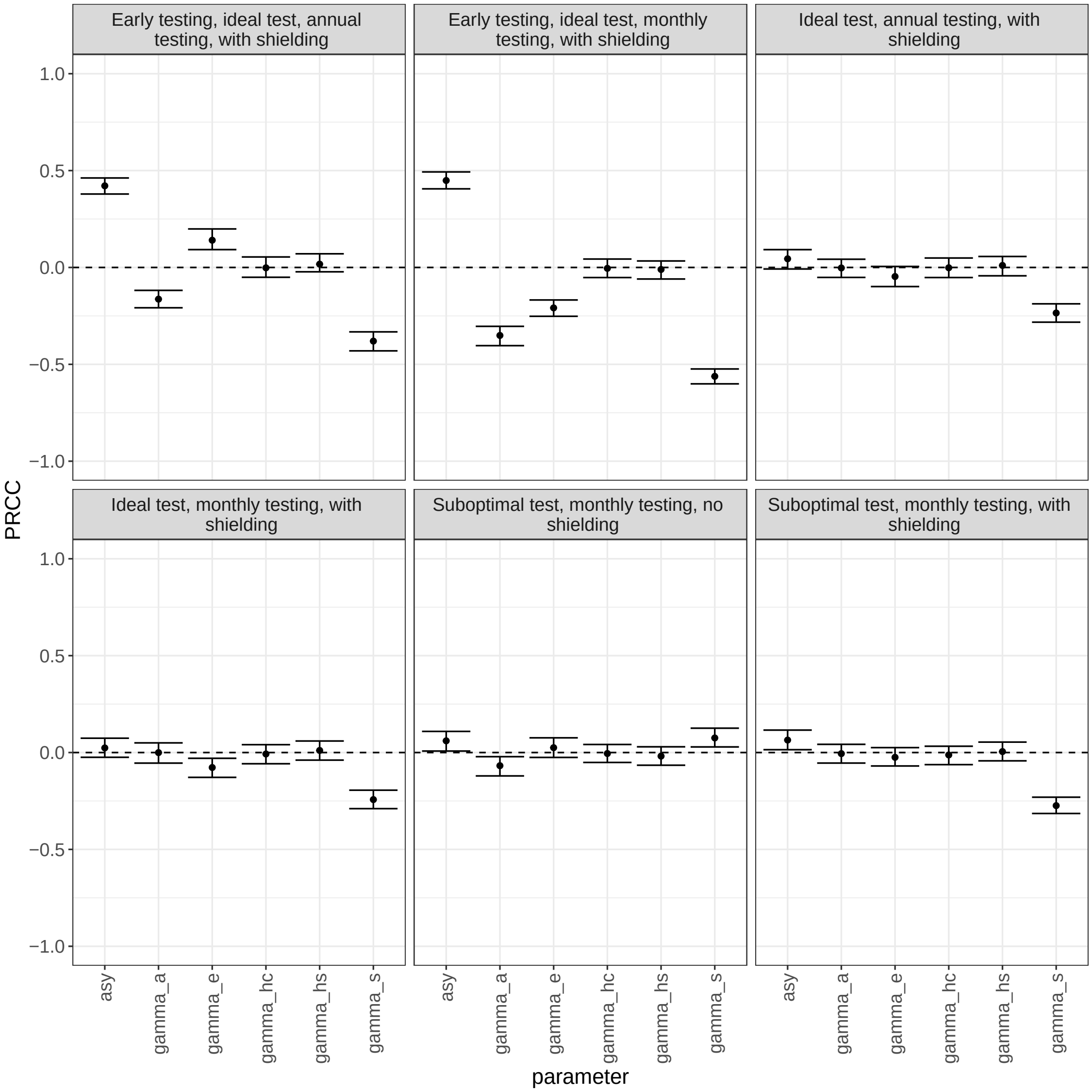

Supplementary Figure 12: **Partial rank correlations coefficients for New York City.** PRCC values are shown for key fixed parameters in the model, including the relative infectiousness of asymptomatic cases (η), the latent (incubation) period (γ_e), recovery rates for both symptomatic and asymptomatic infections (γ_a, γ_s), and duration of hospitalization (γ_{hs}, γ_{hc}).We used Latin Hypercube Sampling to generate 300 random parameter sets based on probable ranges for each parameter, assuming a uniform distribution within each range. Error bars represent the 95% bootstrap confidence intervals (1000 bootstrap replicates) for the Pearson correlation coefficient accounting for multiple (6, one for each parameter tested) comparisons using a Bonferroni correction.

S8. Adding vaccination to the model

In this extension of our main model, we assume a constant complete vaccination rate of 0.5% of the population per day (similar to the US peak distribution of 3 million doses/day when accounting for lower rates due to the need to administer 2 doses to complete the series) starting on January 1, 2021. We assume a vaccine efficacy of 95%: vaccinated, protected individuals (95% of vaccinees) are moved from the untested compartments to the tested, recovered (R) compartment and vaccinated, unprotected persons (5% of vaccinees), are placed in the tested, susceptible (S) compartment. This represents an "all-or-nothing" vaccine, whereby vaccinated persons receive complete protection from SARS-CoV-2 infection and disease. Interestingly, we find that the impact on overall deaths is not large relative to shielding if vaccination is implemented at a rate similar to that seen currently (and surmise that less effective vaccines will also lead to modest reductions in overall fatalities). While vaccines are undoubtedly highly effective at mitigating pandemic effects, this result underscores the large effect of implementing a testing and shielding intervention on a population-level. On top of the impact of a shielding strategy (assuming both high levels of shielding and rapid testing rates), vaccination provides little additional benefit. Of note, in New York City, where the benefit of shielding is very sensitive to small changes in test specificity (Figure 4), our scenarios with vaccination lead to slightly more deaths than those with shielding alone, since a vaccine with $< 100\%$ efficacy leads to 'false positives', and thus increased risk because those who are vaccinated but unprotected resume pre-pandemic levels of social interaction but can still be infected and transmit).

Location	Vaccination and shielding	Shielding only	No intervention
New York City	3505	1766	5069
South Florida	2302	2327	3818
Washington	4256	6233	17528

Supplementary Table 4: Deaths from November 1, 2020-June 1, 2021 under each scenario when adding vaccination to the model. We assume a shielding strategy of monthly testing with a highly-specific test (sp = 0.998) and 5:1 shielding that starts on November 1, 2020

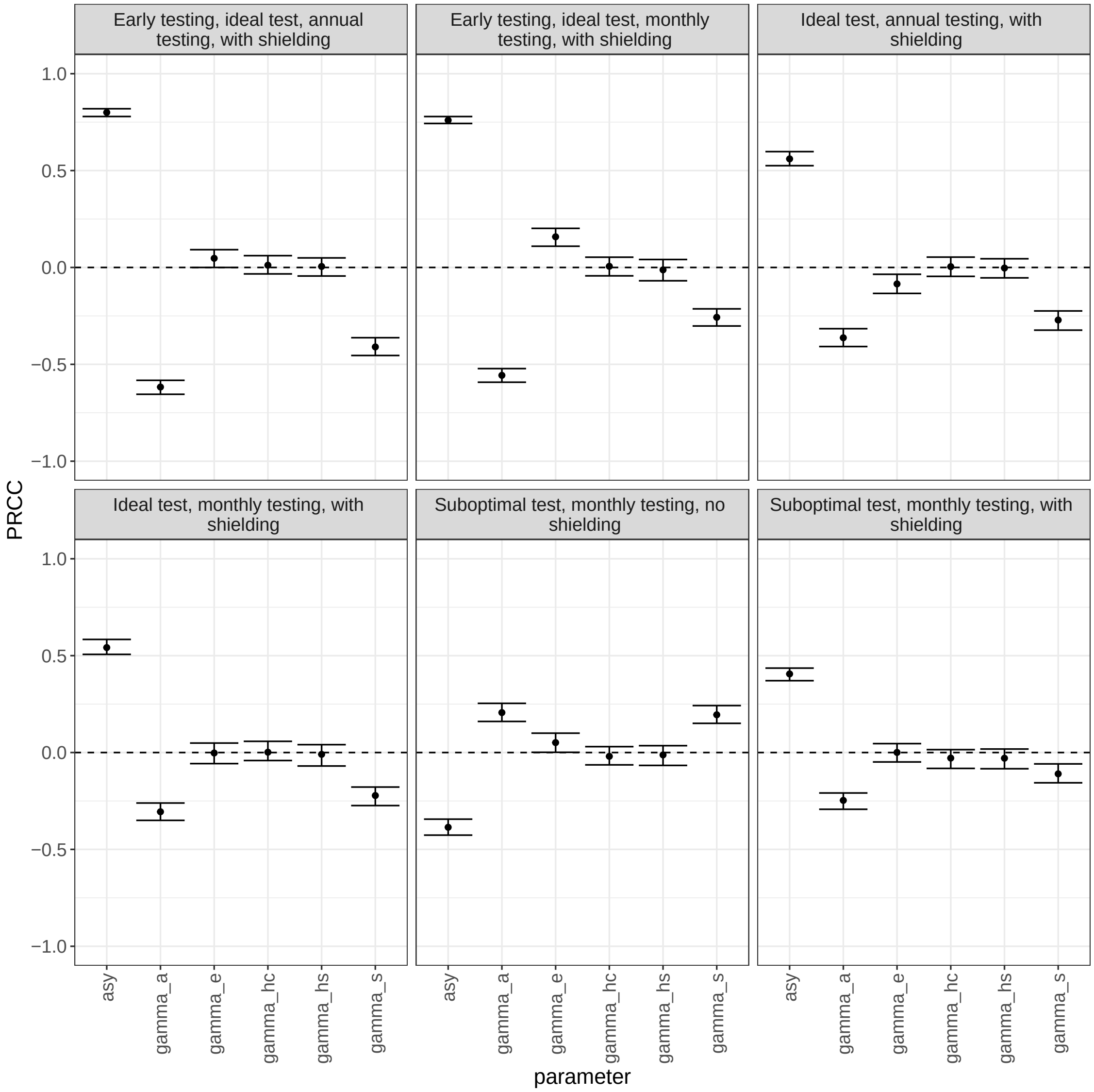

Supplementary Figure 13: **Partial rank correlations coefficients for South Florida.** PRCC values are shown for key fixed parameters in the model, including the relative infectiousness of asymptomatic cases (η), the latent (incubation) period (γ_e), recovery rates for both symptomatic and asymptomatic infections (γ_a, γ_s), and duration of hospitalization (γ_{hs}, γ_{hc}). We used Latin Hypercube Sampling to generate 300 random parameter sets based on probable ranges for each parameter, assuming a uniform distribution within each range.Error bars represent the 95% bootstrap confidence intervals (1000 bootstrap replicates) for the Pearson correlation coefficient accounting for multiple (6, one for each parameter tested) comparisons using a Bonferroni correction.

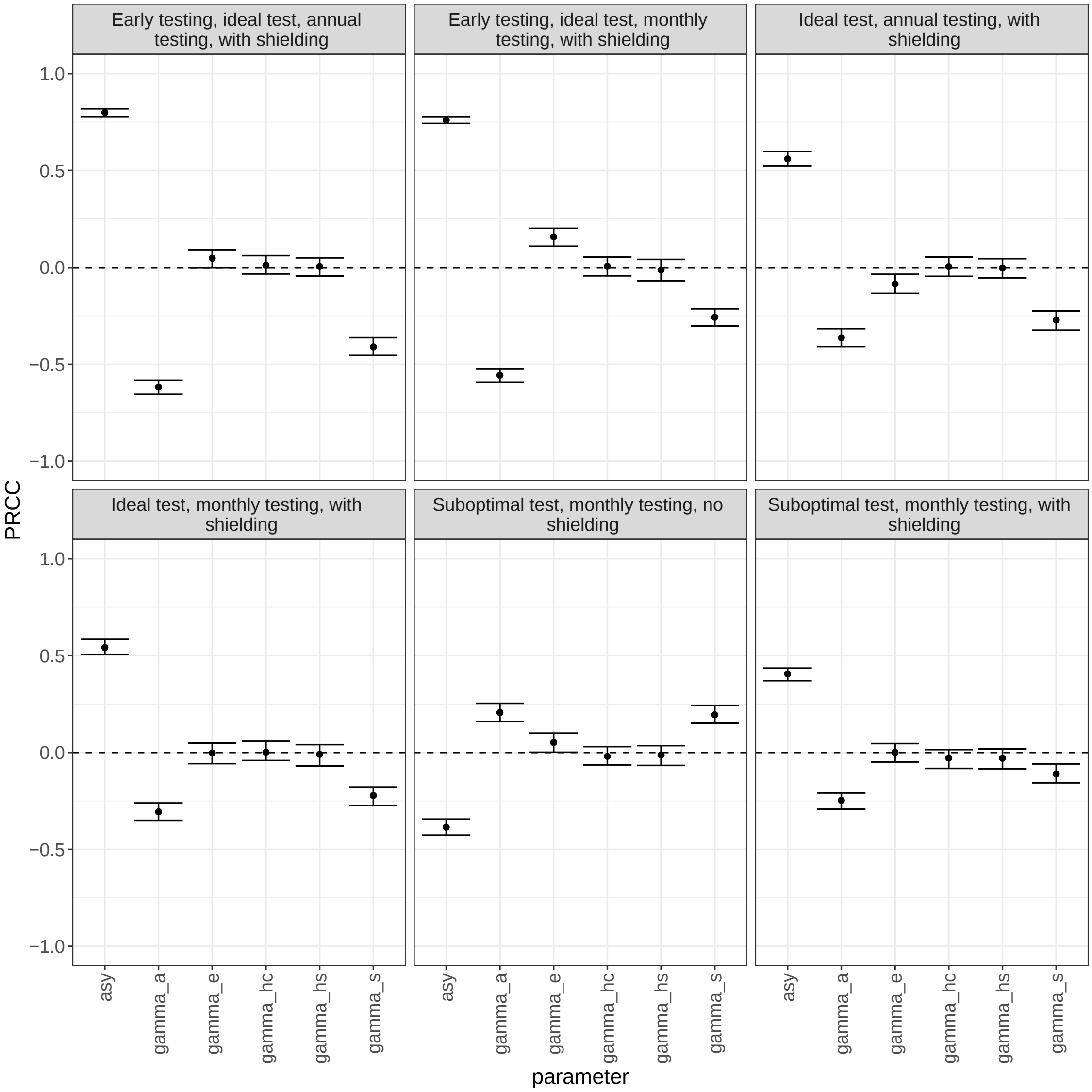

Supplementary Figure 14: **Partial rank correlations coefficients for Washington.** PRCC values are shown for key fixed parameters in the model, including the relative infectiousness of asymptomatic cases (η), the latent (incubation) period (γ_e), recovery rates for both symptomatic and asymptomatic infections (γ_a, γ_s), and duration of hospitalization (γ_{hs}, γ_{hc}). We used Latin Hypercube Sampling to generate 300 random parameter sets based on probable ranges for each parameter, assuming a uniform distribution within each range. Error bars represent the 95% bootstrap confidence intervals (1000 bootstrap replicates) for the Pearson correlation coefficient accounting for multiple (6, one for each parameter tested) comparisons using a Bonferroni correction.

References

[1] Prem K, Cook AR, Jit M. Projecting social contact matrices in 152 countries using contact surveys and demographic data. PLOS Computational Biology. 2017 Sep;13(9):e1005697. Publisher: Public Library of Science. Available from: https://journals.plos.org/ploscompbiol/article?id=10.1371/journal.pcbi.1005697.

[2] Weitz JS, Beckett SJ, Coenen AR, Demory D, Dominguez-Mirazo M, Dushoff J, et al. Modeling shield immunity to reduce COVID-19 epidemic spread. Nature Medicine. 2020;26:849–854. Available from: http://www.nature.com/articles/s41591-020-0895-3.

[3] Havers FP, Reed C, Lim T, Montgomery JM, Klena JD, Hall AJ, et al. Seroprevalence of Antibodies to SARS-CoV-2 in 10 Sites in the United States, March 23-May 12, 2020. JAMA Internal Medicine. 2020 Jul;.

[4] He X, Lau EHY, Wu P, Deng X, Wang J, Hao X, et al. Temporal dynamics in viral shedding and transmissibility of COVID-19. Nature Medicine. 2020 Apr;p. 1–4. Publisher: Nature Publishing Group. Available from: https://www.nature.com/articles/s41591-020-0869-5.

[5] Kissler SM, Tedijanto C, Goldstein E, Grad YH, Lipsitch M. Projecting the transmission dynamics of SARS-CoV-2 through the postpandemic period. Science. 2020 Apr;p. eabb5793. Available from: https://www.sciencemag.org/lookup/doi/10.1126/science.abb5793.

[6] Rees EM, Nightingale ES, Jafari Y, Waterlow N, Clifford S, Pearson CAB, et al. COVID-19 length of hospital stay: a systematic review and data synthesis. BMC Medicine. 2020;(270).

[7] Ferguson N. Report 9: Impact of non-pharmaceutical interventions (NPIs) to reduce COVID-19 mortality and healthcare demand;. Available from: https://www.imperial.ac.uk/media/imperial-college/medicine/sph/ide/gida-fellowships/Imperial-College-COVID19-NPI-modelling-16-03-2020.pdf.

[8] Li R, Pei S, Chen B, Song Y, Zhang T, Yang W, et al. Substantial undocumented infection facilitates the rapid dissemination of novel coronavirus (SARS-CoV2). Science. 2020 Mar;p. eabb3221. Available from: https://www.sciencemag.org/lookup/doi/10.1126/science.abb3221.

[9] CDCMMWR. Severe Outcomes Among Patients with Coronavirus Disease 2019 (COVID-19) in United States, February 12–March 16, 2020. MMWR Morbidity and Mortality Weekly Report. 2020;69. Available from: https://www.cdc.gov/mmwr/volumes/69/wr/mm6912e2.htm.

[10] Zhou F, Yu T, Du R, Fan G, Liu Y, Liu Z, et al. Clinical course and risk factors for mortality of adult inpatients with COVID-19 in Wuhan, China: a retrospective cohort study. The Lancet. 2020 Mar;395(10229):1054–1062. Publisher: Elsevier. Available from: https://www.thelancet.com/journals/lancet/article/PIIS0140-6736(20)30566-3/abstract.

[11] Health CfDaR. EUA Authorized Serology Test Performance. FDA. 2020 May;Publisher: FDA. Available from: https://www.fda.gov/medical-devices/emergency-situations-medical-devices/eua-authorized-serology-test-performance.

[12] Bureau UC. Age and Sex Composition in the United States by County: 2015;. Available from: https://www2.census.gov/programs-surveys/popest/datasets/2010-2019/counties/.

[13] May 2019 OES National Industry-Specific Occupational Employment and Wage Estimates;. Library Catalog: www.bls.gov. Available from: https://www.bls.gov/oes/current/oessrci.htm.

[14] Bureau UC. Age and Sex Composition in the United States: 2018;. Available from: https://www.census.gov/data/tables/2018/demo/age-and-sex/2018-age-sex-composition.html.

[15] Times NY. See how all 50 states are reopening (and closing again);. Available from: https://www.nytimes.com/interactive/2020/us/states-reopen-map-coronavirus.html.

[16] local C. Visualizing the impact of policies on COVID-19 response; 2020. Available from: https://www.covid-local.org/amp/.

[17] Government MDC. Stand up Miami;. Available from: https://www.miamigov.com/Government/Stand-Up-Miami.

[18] Lambert B. A Student's Guide to Bayesian Statistics. SAGE; 2018.
