# Supplementary material for: Modeling serological testing to inform relaxation of social distancing for COVID-19 control
Source: Nat Commun. 2021 Dec 3;12:7063. doi: 10.1038/s41467-021-26774-y (PMC8642547; doi:10.1038/s41467-021-26774-y)
Supplement: Supplementary file 3 — Reporting Summary [file 41467_2021_26774_MOESM3_ESM.pdf]

nature portfolio

Corresponding author(s): Alicia Kraay

Last updated by author(s): 2021-10-8

Reporting Summary

Nature Portfolio wishes to improve the reproducibility of the work that we publish. This form provides structure for consistency and transparency in reporting. For further information on Nature Portfolio policies, see our Editorial Policies and the Editorial Policy Checklist.

Statistics

For all statistical analyses, confirm that the following items are present in the figure legend, table legend, main text, or Methods section.

n/a	Confirmed	
☐	☒	The exact sample size (n) for each experimental group/condition, given as a discrete number and unit of measurement
☐	☒	A statement on whether measurements were taken from distinct samples or whether the same sample was measured repeatedly
☐	☒	The statistical test(s) used AND whether they are one- or two-sided<br>*Only common tests should be described solely by name; describe more complex techniques in the Methods section.*
☒	☐	A description of all covariates tested
☐	☒	A description of any assumptions or corrections, such as tests of normality and adjustment for multiple comparisons
☐	☒	A full description of the statistical parameters including central tendency (e.g. means) or other basic estimates (e.g. regression coefficient) AND variation (e.g. standard deviation) or associated estimates of uncertainty (e.g. confidence intervals)
☐	☒	For null hypothesis testing, the test statistic (e.g. F, t, r) with confidence intervals, effect sizes, degrees of freedom and P value noted<br>*Give P values as exact values whenever suitable.*
☐	☒	For Bayesian analysis, information on the choice of priors and Markov chain Monte Carlo settings
☒	☐	For hierarchical and complex designs, identification of the appropriate level for tests and full reporting of outcomes
☐	☒	Estimates of effect sizes (e.g. Cohen's d, Pearson's r), indicating how they were calculated

Our web collection on statistics for biologists contains articles on many of the points above.

Software and code

Policy information about availability of computer code

Data collection	None.
Data analysis	All models were run in R (version 3.6.2) using the package deSolve (version 1.3.0) and socialmixr (version 0.1.6). Translations of the baseline model are available in MatLab (v 2019a) and Python (v 3.7.3). Fitting and estimation were implemented in MatLab R2019a using the MCMCSTAT package (doi: 10.1007/s11222-006-9438-0). Code used to run models are available at https://github.com/lopmanlab/Serological_Shielding.

For manuscripts utilizing custom algorithms or software that are central to the research but not yet described in published literature, software must be made available to editors and reviewers. We strongly encourage code deposition in a community repository (e.g. GitHub). See the Nature Portfolio guidelines for submitting code & software for further information.

Data

Policy information about availability of data

All manuscripts must include a data availability statement. This statement should provide the following information, where applicable:

- Accession codes, unique identifiers, or web links for publicly available datasets
- A description of any restrictions on data availability
- For clinical datasets or third party data, please ensure that the statement adheres to our policy

Death data used for model calibration are available from https://usafacts.org/visualizations/coronavirus-covid-19-spread-map/ and policy data that was used to define the reopening policies by site are available at https://github.com/nytimes/covid-19-data. Seroprevalence data was previously published by Havers et al (16). All data used directly in model fitting are available in the Github repository, specifically at https://github.com/lopmanlab/Serological_Shielding/MCMC_CODE/Matlab/INPUTS.

Field-specific reporting

Please select the one below that is the best fit for your research. If you are not sure, read the appropriate sections before making your selection.

☐ Life sciences ☒ Behavioural & social sciences ☐ Ecological, evolutionary & environmental sciences

For a reference copy of the document with all sections, see nature.com/documents/nr-reporting-summary-flat.pdf

Behavioural & social sciences study design

All studies must disclose on these points even when the disclosure is negative.

Study description	We carried out a mathematical modeling study using publicly available epidemiologic surveillance data.
Research sample	Deaths data on data on reopening policies in the U.S. was sussed to build models of COVID-19 transmission. Data and models represent four metropolitan areas in the U.S. (Washington Puget Sound, New York Metro Area, South Florida) that experienced different trajectories and severity of the COVID-19 pandemic. The counties combined for each of these areas were chosen to match the Havers et al. seroprevalence data.
Sampling strategy	Death data included all COVID-19 deaths in each of the included counties in each location. The seroprevalence study (Havers et al.) used convenience sampling based on all serum samples that had previously been collected for other purposes at two laboratories.
Data collection	Data was collected without a specific hypothesis in mind as a part of routine surveillance activities by local public health agencies (deaths) and for other routine healthcare uses (serology). Therefore, blinding is not relevant to the study question.<br><br>Death time series data is collected by the New York Times from state and local governments and health departments in an attempt to provide a complete record of the ongoing outbreak. ach row of data reports the cumulative number of coronavirus cases and deaths based on our best reporting up to the moment we publish an update. Our counts include both laboratory confirmed and probable cases using criteria that were developed by states and the federal government.<br><br>Sera were tested at CDC in a two-step process, a screening assay followed by a confirmatory assay for presumptive reactive specimens identified through screening. The CDC developed and validated enzyme-linked immunosorbent assay (ELISA) that was used as the confirmatory assay. A specimen was considered reactive if, on confirmatory testing, at a background corrected optical density (OD) of 0.4 and at a serum dilution of 1:100, it had a signal to threshold ratio of >1. The screening assay was similar to the confirmatory assay. Sera were screened at a 1:100 dilution using a qualitative pan immunoglobulin (Ig) ELISA against the pre-fusion stabilized ectodomain of the SARS-CoV-2 spike protein (S). See Havers et al. for more information.
Timing	The death time series includes all reported deaths from March 1, 2020-July 31, 2020. Seroprevalence estimates are based on cross-sectional data collected at different times for each of the three sites. For New York City and Washington, data were collected from March 23-April 1, 2020. For South Florida, data were collected from April 6-10, 2020.
Data exclusions	No data were excluded from the analysis.
Non-participation	As the death and seroprevalence data were both collected through routine surveillance activities, there is no bias due to non-participation.
Randomization	As the death and seroprevalence data were both collected through routine surveillance activities, no randomization was used in this study.

Reporting for specific materials, systems and methods

We require information from authors about some types of materials, experimental systems and methods used in many studies. Here, indicate whether each material, system or method listed is relevant to your study. If you are not sure if a list item applies to your research, read the appropriate section before selecting a response.

Materials & experimental systems

n/a	Involved in the study	
☒	☐	Antibodies
☒	☐	Eukaryotic cell lines
☒	☐	Palaeontology and archaeology
☒	☐	Animals and other organisms
☒	☐	Human research participants
☒	☐	Clinical data
☒	☐	Dual use research of concern

Methods

n/a	Involved in the study	
☒	☐	ChIP-seq
☒	☐	Flow cytometry
☒	☐	MRI-based neuroimaging
